# X-ray absorption linear dichroism at the Ti *K*-edge of rutile (001) TiO_2_ single crystal

**DOI:** 10.1107/S160057752000051X

**Published:** 2020-02-14

**Authors:** T. C. Rossi, D. Grolimund, O. Cannelli, G. F. Mancini, C. Bacellar, D. Kinschel, J. R. Rouxel, N. Ohannessian, D. Pergolesi, M. Chergui

**Affiliations:** a Laboratory of Ultrafast Spectroscopy, Ecole Polytechnique Fédérale de Lausanne SB-ISIC-LSU and Lausanne Centre for Ultrafast Science (LACUS), CH-1015 Lausanne, Switzerland; b Laboratory for Femtochemistry – MicroXAS Beamline Project, Paul Scherrer Institute, CH-5232 Villigen, Switzerland; c Laboratory for Multiscale Materials Experiments, Paul Scherrer Institute, CH-5232 Villigen, Switzerland; d Electrochemistry Laboratory, Paul Scherrer Institute, CH-5232 Villigen, Switzerland

**Keywords:** rutile TiO_2_, linear dichroism, X-ray absorption spectroscopy, finite difference method

## Abstract

X-ray absorption linear dichroism of rutile TiO_2_ at the Ti *K*-edge provides information about the electronic states involved in the pre-edge transitions. Here, linear dichroism with high energy resolution is analyzed in combination with *ab initio* finite difference method calculations and spherical tensor analysis. It provides an assignment of the three pre-edge peaks beyond the octahedral crystal field splitting approximation and estimates the spatial extension of the corresponding final states. It is then discussed for the first time the X-ray absorption (XAS) of pentacoordinated titanium atoms due to oxygen vacancies and it is found that, similarly to anatase TiO_2_, rutile is expected to exhibit a transition on the low-energy side of peak A3. Its apparent absence in the experiment is related to the degree of *p*–*d* orbital mixing which is small in rutile due to its centrosymmetric point group. A recent XAS linear dichroism study on anatase TiO_2_ single crystals has shown that peak A2 has an intrinsic origin and is due to a quadrupolar transition to the 3*d* energy levels. In rutile, due to its centrosymmetric point group, the corresponding peak A2 has a small dipole moment explaining the weak transition. The results are confronted with recent picosecond X-ray absorption spectroscopy on rutile TiO_2_ nanoparticles.

## Abstract 2

Linear dichroism at the Ti *K*-edge is investigated in rutile TiO_2_ single crystal. A complete assignment of the pre-edge is provided based on finite difference method calculations and spherical tensor analysis. The origin of weak peak A2 similar to anatase TiO_2_ is discussed from the presence of oxygen vacancies or from an intrinsic quadrupolar transition.

## 1. Introduction   

**Figure 1:**
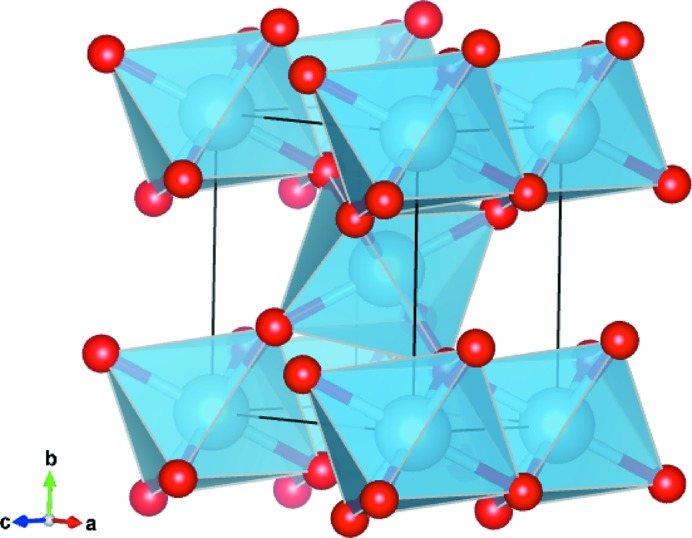
Conventional unit cell of rutile TiO_2_. Two equivalent Ti sites are present in the unit cell at the corners and in the centre which correspond to different orientations of the TiO_6_ octahedra.

Titanium dioxide (TiO_2_) is a wide-band-gap insulator which exhibits superior properties in a wide range of applications (Liu & Aydil, 2009 ▸; Miyoshi *et al.*, 2018 ▸; Gao *et al.*, 2019 ▸). Its natural polymorph in bulk crystals is rutile TiO_2_ (r-TiO_2_) in which TiO_6_ octahedra with *D*_2*h*_ point group symmetry are connected to ten neighbouring octahedra via a corner or an edge (Fig. 1 ▸). Anatase is another polymorph of TiO_2_ (a-TiO_2_) which becomes the most abundant phase in nanomaterials. It is also made of TiO_6_ octahedra but in a different spatial arrangement which generates an indirect band gap, contrary to rutile. Although a-TiO_2_ exhibits more appealing properties in a lot of applications such as photocatalysis (Linsebigler *et al.*, 1995 ▸), the two polymorphs exhibit similar performances in photovoltaics (Park *et al.*, 2000 ▸).

Optoelectronic device performances are in part governed by the mobility of photogenerated charge carriers. The mobility can be reduced by coupling with the lattice modes, generating heavier quasiparticles which can localize in a few unit cells and slow down their recombination (Okamoto *et al.*, 2010 ▸, 2011 ▸). Anatase TiO_2_ is prone to lattice deformation which generates self-trapped excitons under band gap excitation (Tang *et al.*, 1995 ▸) while free excitons and bound excitons are observed in the photoluminescence spectra of rutile (Amtout & Leonelli, 1992 ▸). Recent progress in the description of the bound excitons spatial extension (Baldini *et al.*, 2017*a* ▸,*b* ▸) has shown that experimental techniques which are more sensitive to the local character of photogenerated quasiparticles are required to obtain the fine details of their geometrical and electronic structure.

XAS at the transition metal *K*-edge is an element-specific spectroscopy technique which can access both the electronic structure and local geometry with subatomic spatial resolution (Rehr & Albers, 2000 ▸). It is usually decomposed into three spectral regions around the absorption edge which correspond to the absorption of the X-ray photon by an electron in the 1*s* orbital: (i) the extended X-ray absorption fine structure (EXAFS) at >50 eV above the edge corresponds to single scattering events of the photoemitted electron by surrounding atoms – it provides information about the bond distances and is straightforward to interpret (Hollas, 2004 ▸); (ii) the X-ray absorption near-edge structure (XANES) at the absorption edge up to ∼50 eV above it corresponds to multiple scattering (MS) events of the photoemitted electrons which provide information about bond angles and distances. XANES is usually more complicated to interpret because of the contribution of transitions to the continuum and below to diffuse 4*p* orbitals, as well as to MS; (iii) the pre-edge exhibits weak features below the absorption edge which are due to transitions to bound states above the Fermi level. These transitions can have quadrupolar and/or dipolar character depending on the selection rules and the local symmetry around the atom absorbing the X-radiation. Since the pioneering work of Brümmer and Dräger, it is established that the dipole and quadrupolar matrix elements of the transitions can be separated with linear dichroism (LD) XAS experiments in which different crystal orientations are probed by the electric field and wavevectors of the X-rays (Brümmer *et al.*, 1971 ▸; Dräger *et al.*, 1988 ▸). More recent methods such as resonant X-ray emission spectroscopy (Szlachetko *et al.*, 2014 ▸) or resonant inelastic X-ray scattering (Bagger *et al.*, 2017 ▸) provide the same information since they involve the XAS matrix elements. Quadrupolar transitions are usually 10–100 times weaker than dipolar ones which makes them difficult to study experimentally with standard LD XAS techniques as used in this work. In this respect, the Borrmann effect can be used to enhance the amplitude of the quadrupole transitions (Pettifer *et al.*, 2008 ▸; Tolkiehn *et al.*, 2011 ▸).

The XAS of r-TiO_2_ at the *K*-edge exhibits three pre-edge peaks labelled A1, A3 and B in this work.^1^ Previous XAS measurements have shown a pronounced LD of the pre-edge features especially for peak A1 (Brouder *et al.*, 1990 ▸) which undergoes a 90°-periodic 30% amplitude variation when the sample is rotated in the ([100], [010]) plane indicating a quadrupolar component. A pronounced LD is also observed in the XANES (Poumellec *et al.*, 1991 ▸) when the sample is rotated in the ([100], [001]) plane where components along the ordinary and extraordinary axes are exchanged (Poumellec *et al.*, 1995 ▸). The LD of peaks A1 and A3 has been confirmed more recently as well as the quadrupolar nature of peak A1 by angular-resolved resonant Auger spectroscopy (Le Fèvre *et al.*, 2005 ▸). We have shown how the analysis of the LD can assign final states to the bound transitions in the pre-edge of a-TiO_2_ (Rossi *et al.*, 2019 ▸). In particular, for distorted octahedral systems such as a-TiO_2_ and r-TiO_2_, the usual crystal field splitting into *t*_2*g*_ and *e*_*g*_ final states is inappropriate and does not account for the experimental evolution of the peak amplitudes with incident electric field and wavevector (Ruiz-Lopez & Munoz-Paez, 1991 ▸). In this paper, we analyze the LD of r-TiO_2_ (001) single crystal which provides an assignment of the final states involved in each pre-edge transition in the monoelectronic approximation. The novelty of the approach resides in the combination of *ab initio* and analytical theoretical techniques to infer the presence of given orbitals in the final states involved in the pre-edge transitions.

The assignment of the final states involved in the pre-edge goes hand in hand with theoretical modelling. Muffin-tin MS calculations are insufficient to describe the XAS of anisotropic materials which require full potential methods (Joly *et al.*, 1999 ▸; Bunău & Joly, 2009 ▸; Kravtsova *et al.*, 2010 ▸). The finite difference method (FDM) developed by Y. Joly (Joly *et al.*, 1999 ▸; Joly, 2001 ▸) has shown that, in r-TiO_2_, A1 is a quadrupolar transition to *t*_2*g*_ orbitals, A3 is essentially dipolar with a weak *e*_*g*_ quadrupolar contribution, and B is purely dipolar (Cabaret *et al.*, 1999 ▸). The spatial extension of the final state can be used to address the dipolar or quadrupolar character of the transition. We have shown that the quadrupole transitions in a-TiO_2_ populate a final state localized in the TiO_6_ octahedron where the Ti atom absorbs the X-radiation (Rossi *et al.*, 2019 ▸). Final states which result from on-site or off-site *p*–*d* orbital hybridization can have various spatial extensions sensitive to the first coordinating shell of TiO_6_ octahedra or even further away. In this work, we investigate the spatial extension of the final states in detail using *ab initio* FDM calculations.

The pre-edge of a-TiO_2_ and r-TiO_2_ differs by the presence of a pronounced A2 peak on the low-energy side of peak A3 in a-TiO_2_ which is absent in r-TiO_2_. Empirical studies have shown that the amplitude of A2 is related to the amount of oxygen vacancies in a-TiO_2_ while no difference is observed in the r-TiO_2_ case (Luca *et al.*, 1998 ▸; Hanley *et al.*, 2002 ▸; Luca, 2009 ▸). Recent theoretical modelings have shown that the local structural disorder also plays an important role in the amplitude of A2 in a-TiO_2_ (Zhang *et al.*, 2008 ▸; Triana *et al.*, 2016 ▸). Our recent LD study has shown that peak A2 is present in a-TiO_2_ single crystals and is due to an intrinsic quadrupolar transition which exhibits LD (Rossi *et al.*, 2019 ▸). It is the result of the weak *p*–*d* hybridization and *p*-density of states (DOS). *Ab initio* calculations including the explicit treatment of the core-hole interaction in the Bethe–Salpeter equation are in agreement with this assignment (Vorwerk *et al.*, 2017 ▸) and suggest that peak A2 may be seen in r-TiO_2_ on the low-energy side of peak A3 under azimuthal rotation (Shirley, 2004 ▸). The final states of peaks A1 and A2 in a-TiO_2_ have orthogonal *p*-DOS contributions which indicate a difference in the binding energy with the core-hole. The energy splitting between these bound states has a similar origin to the bound excitonic states found in a-TiO_2_ in the optical range (Baldini *et al.*, 2017*a* ▸; Chiodo *et al.*, 2010 ▸). It is thus surprising *a priori* that the A2 peak is not observed in the r-TiO_2_ polymorph either due to an intrinsic quadrupolar transition in the bulk or because of penta-coordinated Ti atoms due to oxygen vacancies. This is clarified in this work as we show that the degree of *p*–*d* hybridization is key to the amplitude of peak A2 in both cases.

In regard to the discrepancy of orbital assignment and spatial extension of the final state in previous studies as well as the absence of discussion about the absence of peak A2 in r-TiO_2_, a combined experimental and theoretical LD XAS study is valuable to provide an accurate description of the orbital content in the pre-edge. In this work, we combine the measured XAS at different incidence angles at the surface of (001) r-TiO_2_ with FDM calculations and spherical harmonic analysis (Brouder *et al.*, 2008 ▸) to provide an unambiguous assignment beyond the usual octahedral crystal field splitting approximation which is inappropriate for r-TiO_2_ (Ruiz-Lopez & Munoz-Paez, 1991 ▸). Previous assignments of the pre-edge peaks are provided in Table 1 ▸ together with the ones established in this work.

## 2. Experimental   

The LD measurements were performed at the MicroXAS beamline of the SLS in Villigen, Switzerland, using a double Si(311) crystal monochromator to optimize the energy resolution. Energy calibration is performed from the first derivative of the XAS of a thin Ti foil. We used a moderately unfocused rectangular-shaped X-ray beam of 20 µm (horizontal) × 200 µm (vertical). The XAS is obtained in total fluorescence yield with a Ketek Axas detector system with Vitus H30 SFF and ultralow capacitance Cube-Asic preamplifier (Ketek GmbH).

**Figure 2:**
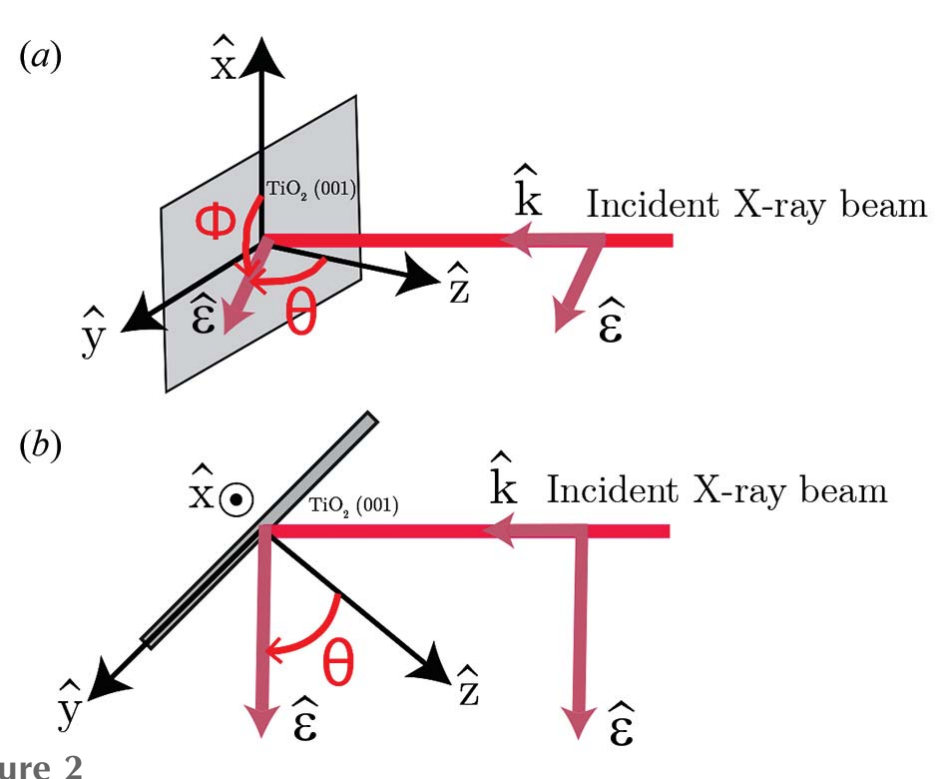
Linear dichroism experimental geometry and angular conventions with (*a*) side view and (*b*) top view. The sample surface is in grey while the incident X-ray beam is in pink. A set of Euler angles (θ, ϕ, ψ) is used to orient the electric field 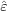 and wavevector 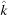 of the incident X-ray beam with respect to the sample.

The sample consists of a (001)-oriented crystalline r-TiO_2_ thin film [thickness 46 nm; see §S1 of the supporting information (SI) for details on the sample growth and characterizations] mounted on a set of translation and rotation stages allowing a fixed centre of rotation at the sample surface. By convention, a set of Euler angles (θ, ϕ, ψ) orients the electric field 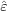 and wavevector 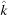 with respect to the sample crystallographic axes (Fig. 2 ▸). θ measures the angle between 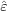 and the [001] crystal direction (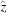 axis of the sample frame) orthogonal to the surface. ϕ measures the angle between 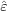 and the sample rotation axis 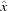. In principle, a third angle ψ is necessary to fix the position of the wavevector in the orthogonal plane to the electric field but here ψ = 0°. The θ angles reported in the experimental datasets are with a maximum systematic offset of ±0.2° which comes from the precision setting up the θ = 0° reference from the sample half-clipping of the X-ray beam at grazing incidence. The precision of the rotation stage of ±0.01° is negligible with respect to this angular offset.

LD is usually studied with the sample rotated in the plane orthogonal to the incident X-ray beam (ϕ-rotation) (Brouder *et al.*, 1990 ▸). Here, the sample is rotated around 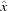 (θ-rotation) where the changes in the XAS are the most pronounced. Under this rotation, the X-ray footprint onto the sample surface changes with θ. However, we clearly show that this procedure does not introduce spectral distortions because the effective penetration depth of the X-rays through the material [between ∼85 nm and ∼540 nm across the absorption *K*-edge of r-TiO_2_ for the largest footprint at θ = 1° used here (Henke *et al.*, 1993 ▸)] is kept constant as the sample is thinner than the attenuation length at the Ti *K*-edge. Instead, the total amount of material probed by the X-rays changes due to the larger X-ray footprint when θ increases and a renormalization over the number of detected X-ray fluorescence photons is required. This is done with the support of finite difference method near-edge structure (FDMNES) calculations (*vide infra*) since a few energy points have θ-independent cross-sections as previously reported on other systems (Frétigny *et al.*, 1986 ▸; Stizza *et al.*, 1986 ▸; Oyanagi *et al.*, 1987 ▸, 1989 ▸; George *et al.*, 1989 ▸; Loupias *et al.*, 1990 ▸; Pettifer *et al.*, 1990 ▸; Rossi *et al.*, 2019 ▸).^2^ With this renormalization procedure performed at a single energy point (4987.3 eV), we could obtain a set of experimental points with θ-independent cross-sections at the energies predicted by the theory confirming the reliability of the method (*vide infra*). Hence, crystalline thin films with suitable thicknesses with respect to the X-ray penetration depth offer more possibilities to study LD effects than single crystals and prevent the usual self-absorption distortion of bulk materials using total fluorescence yield detection (Carboni *et al.*, 2005 ▸).

### 2.1. FDM calculations   

The *ab initio* calculations of the XAS cross-section were performed with the full potential finite difference method (FDM) as implemented in the *FDMNES* package (Joly *et al.*, 1999 ▸; Joly, 2001 ▸). A cluster of 7.0 Å was used for the calculation with the fundamental electronic configuration of the oxygen atom and an excited state configuration for the titanium atom (Ti:[Ar]3*d* ^1^4*s* ^2^4*p* ^1^). Previous calculations have shown that it converges faster to the same results as the starting electronic structure 3*d* ^2^4*s* ^2^ showing the robustness of the method (Bunău & Joly, 2009 ▸). We checked the convergence of the calculation for increasing cluster sizes and found minor evolution for larger cluster radii than 7.0 Å. The Hedin–Lundqvist exchange-correlation potential is used (Hedin & Lundqvist, 2001 ▸) although it provides similar results as the other widely used Perdew–Wang potential (Perdew & Wang, 1992 ▸). The agreement between the experimental and theoretical pre-edge feature energies can be better matched by adding self-consistency to the calculation which acts especially on the 3*d* states close to the Fermi level, decreasing the energy of peak A1 (Joly *et al.*, 2009 ▸). Alternatively, the change in the screening of the 3*d* electrons of titanium provides the same effect decreasing the energy of quadrupole allowed transitions. This is the method we have used by considering a screening of 0.85 for the 3*d* electrons of Ti similar to previous studies (Aïfa *et al.*, 1997 ▸). An arctan convolution with maximum broadening of 1.5 eV is applied to the calculated spectra to account for core-hole lifetime and a constant broadening of 0.095 eV is applied to account for the experimental resolution.

### 2.2. Spherical tensor analysis of the dipole and quadrupole cross-sections   

Analytical expressions of the dipole and quadrupole XAS cross-sections [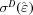 and 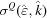, respectively] are obtained from their expansion into spherical harmonic components (Brouder, 1990 ▸; Brouder *et al.*, 1990 ▸). The expressions of 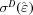 and 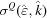 depend on the crystal point group which is *D*_2*h*_ (2/*mmm*) for r-TiO_2_. For the dipole cross-section we obtain
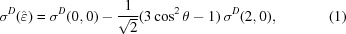
and, for the quadrupole cross-section,
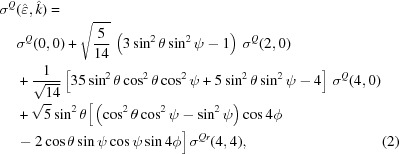
where (θ, ϕ, ψ) are Euler angles in the Ti point group frame. σ^*X*^(*l*, *m*) with *X* = *D*, *Q* is the spherical tensor with rank *l* and projection *m*. The Euler angles (θ, ϕ, ψ) in the experiment are referenced to the crystal frame which is rotated with respect to the Euler angles in the Ti site frame. Consequently, the angles in equations (1) and (2) differ from the angles defined in Fig. 2 ▸ by rotation around θ and ϕ. For instance, the 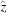 axis of the site frame is rotated by an angle π/2 around a bisecting axis in the 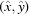 plane of the site frame with respect to the crystal frame. Consequently, the formulas (1) and (2) need to be modified to match the incidence angle θ used in the experiment [details in §S7 (SI)]. The dipole cross-section is now given by 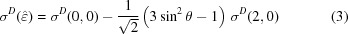
and the quadrupole cross-section by
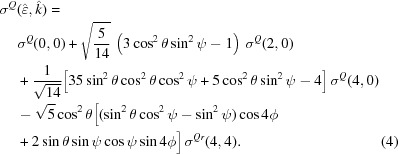
Although some terms of 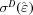 and 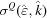 may be negative, the total dipolar and quadrupolar cross-sections must be positive putting constraints on the values of σ^*D*^(*l*, *m*) and σ^*Q*^(*l*, *m*). The electric field 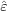 and wavevector 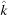 coordinates in the 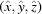 basis of Fig. 2 ▸ are given by
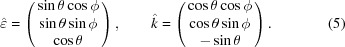
Hence the detail of the XAS cross-section angular dependence requires an estimate of the spherical tensors σ^*D*^(*l*, *m*) and σ^*Q*^(*l*, *m*) as performed elsewhere (Brouder *et al.*, 2008 ▸). The XAS measured experimentally is an average over equivalent Ti atoms under the symmetry operations of the crystal point group. The analytical formula representing this average requires the site symmetrization and crystal symmetrization of the spherical tensors, which is provided in §S7 and §S7.2 (SI). In the case of θ-rotation at ϕ = 0° described in this paper, the two equivalent Ti sites have the same XAS spectra. We provide spectra calculated at the two equivalent Ti sites of r-TiO_2_ under θ [Fig. S8 (SI)] and ϕ rotation [Fig. S9 (SI)] showing this effect. Assuming pure 3*d* and 4*p* final states in the monoelectronic approximation, analytical expressions are provided for 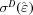 and 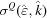 whose angular dependence with θ are given in Table 2 ▸. The full expressions of the cross-sections are provided in §S7.4 (SI). In this paper, we analyze the angular dependence of the pre-edge peak intensities with θ and assign them to specific final states corresponding to Ti-3*d* and/or 4*p* orbitals with the support of both FDM and spherical tensor analysis.

## 3. Results   

**Figure 3:**
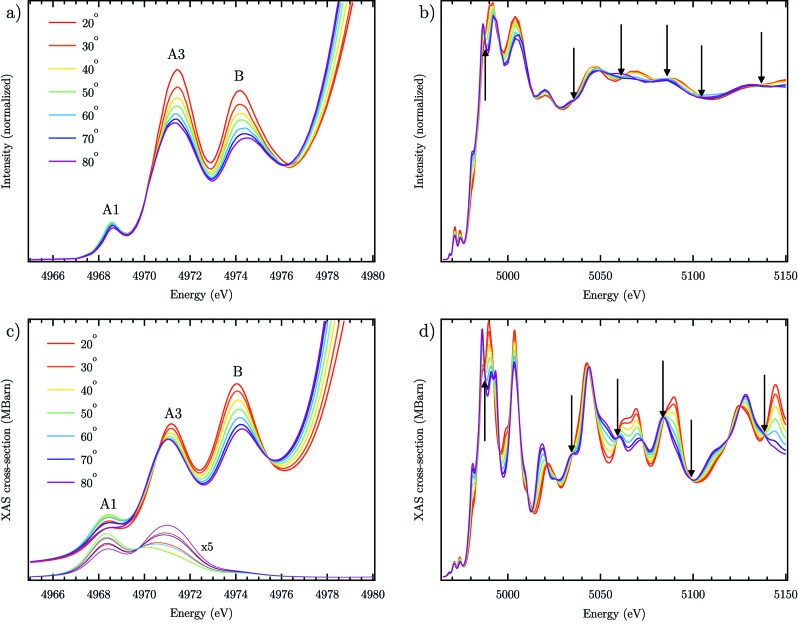
(*a*), (*b*) Experimental and (*c*), (*d*) theoretical evolution of the XAS at the Ti *K*-edge of r-TiO_2_ for different angles of incidence θ. (*a*), (*c*) Pre-edge region. (*b*), (*d*) XANES and EXAFS. Isosbestic points are shown with black arrows. Thick lines in (*c*) are spectra calculated with dipole and quadrupole matrix elements; thin lines show the quadrupole matrix elements contribution only.

The experimental evolution of the normalized XAS with angle θ is depicted in Fig. 3(*b*) ▸ with an emphasis on the pre-edge given in Fig. 3(*a*) ▸. The results are similar to previous published spectra under similar rotation between [001] and 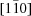 (Aïfa *et al.*, 1997 ▸). In the pre-edge, the amplitudes of peaks A3 and B are notably affected by the sample orientation with a red shift of the maximum for peak A3 and a blue shift for peak B with θ. Strong changes are also observed in the post-edge region showing that the LD remains well above the edge. Calculated spectra using *FDMNES* are shown in Figs. 3(*c*) and 3(*d*) ▸ with the same polarization conditions as in the experiment. The evolution of the peak B amplitude and energy shift is nicely reproduced while a red shift with θ appears for peak A3 with an underestimated amplitude change. The ratio between A3 and B amplitudes changes between the experiment and the theory [Figs. 3(*a*) and 3(*c*) ▸] similarly to previous calculations with the same computational method (Joly *et al.*, 1999 ▸) but also with full multiple scattering (Zhang *et al.*, 2008 ▸). The evolution of peak A1 is weak in accord with the experiment and with previous results which assigned it to a purely quadrupolar transition (Uozumi *et al.*, 1992 ▸; Parlebas, 1993 ▸; Parlebas *et al.*, 1995 ▸). An excellent reproduction of the experimental data is also obtained above the edge where the isosbestic points are found in agreement, marked by black arrows in Fig. 3 ▸. This shows the absence of self-absorption effects in the experimental data which would distort the spectra (Carboni *et al.*, 2005 ▸).

**Figure 4:**
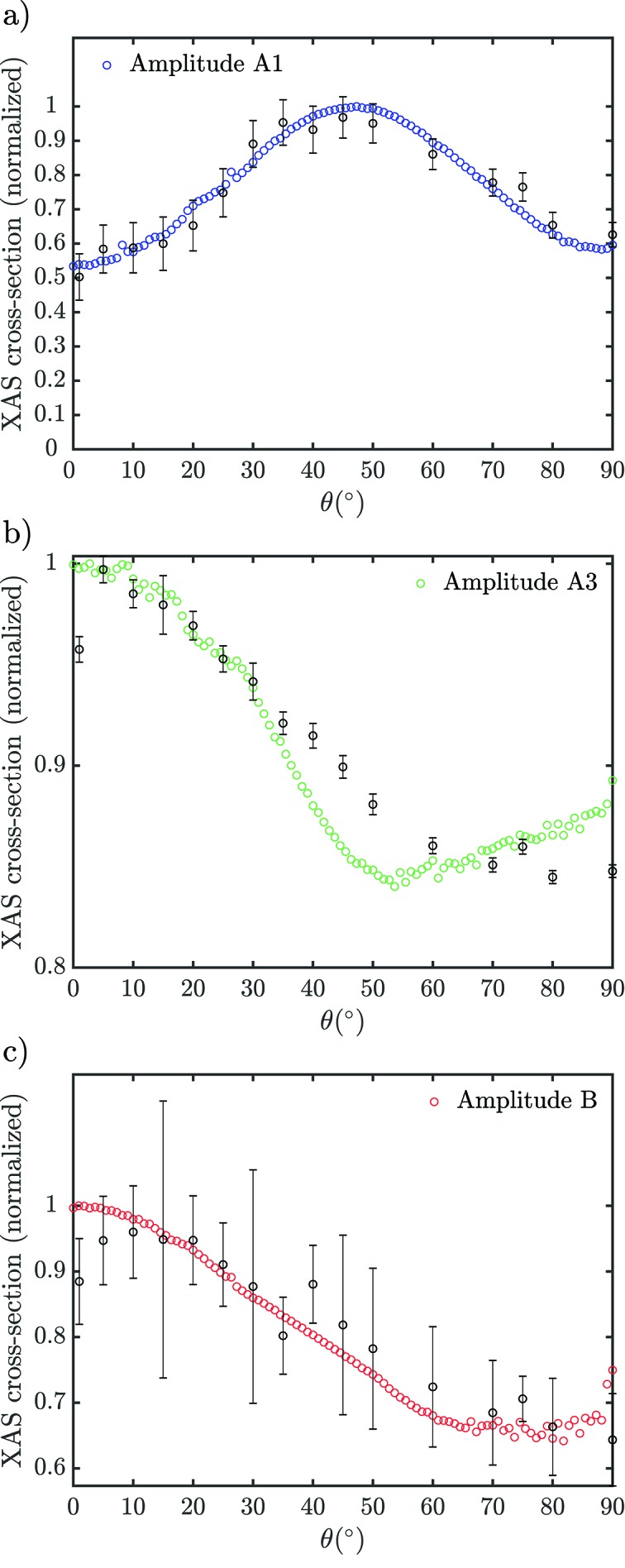
Evolution of the experimental (black circles with error bars) and theoretical (coloured circles) amplitudes of the pre-edge peaks (*a*) A1, (*b*) A3 and (*c*) B in r-TiO_2_ going from an electric field along [001] (θ = 0°) to [100] (θ = 90°). The error bars represent 95% confidence interval in the fitted experimental amplitude. The sum of dipole and quadrupole components is fitted in the theoretical data. Details about the fitting procedure and the fitting results are given in §S2 and §S3 (SI).

We have fitted the pre-edge peaks with Gaussians for the experimental data and with pseudo-Voigt functions for the theoretical data in order to extract the evolution of the amplitude with the incidence angle θ. The fitted amplitudes of the three experimental pre-edge edge peaks appear as black circles with error bars in Fig. 4 ▸. The vertical scale is modified to match the theoretical evolution of the XAS cross-section according to a procedure described in §S2 (SI). Fittings to individual spectra are given in Fig. S4 (SI). Peak A1 undergoes an almost pure quadrupolar evolution with 90°-periodicity, reaching its maximum amplitude for θ = 45° [Fig. 4(*a*) ▸]. However, we notice a slight deviation to this behaviour with the cross-section being larger when θ → 90° than when θ → 0°. This behaviour is in excellent agreement with the theory [blue circles in Fig. 4(*a*) ▸] which indicates a weak dipolar contribution underlying the overwhelming quadrupolar one. Experimentally, both peaks A3 and B have a monotonic decrease with θ which does not match the theory for peak A3 in which an increase in amplitude appears from θ ≃ 60° [Figs. 4(*b*) and 4(*c*) ▸]. This is likely due to the underestimated amplitude of peak A3 and its evolution which competes with peak broadening changes with the incidence angle.

In order to assign the 3*d* and 4*p* orbitals contributing to the final state of the pre-edge transitions, we look at the DOS in the pre-edge provided by *FDMNES* [Fig. S7 (SI)] as well as the expected evolution from the spherical tensor analysis (Table 2 ▸).

In the region of peak A1, the *p*-DOS is negligible [Fig. S7(*b*) (SI)] while the DOS of all possible *d*-states is present except *d*_*yz*_ [Fig. S7(*c*) (SI)]. This is in agreement with the dominant quadrupolar evolution observed experimentally [Fig. 4(*a*) ▸], compatible with 3*d* orbitals contributing to the final state. Similar results were obtained by others under ϕ-rotation (Parlebas, 1993 ▸; Parlebas *et al.*, 1995 ▸). The 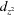 and *d*_*xy*_-DOS is ∼500 times larger than the *d*_*xz*_, *d*_*yz*_-DOS [Fig. S7(*c*) (SI)]. The spherical tensor analysis of the cross-section involving 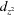 and *d*_*xy*_ orbitals in the final state gives a maximum for θ = 45° [Fig. S6(*b*) (SI)], in agreement with the experiment [Fig. 4(*a*) ▸] and the calculated DOS [Fig. S7(*c*) (SI)]. The deviation from the ideal quadrupolar evolution goes in the direction of a weak hybridization with 4*p*_*x*,*y*_ states which reach their maximum amplitude for θ = 90° according to the spherical tensor analysis [Fig. S6(*a*) (SI)].

The experimental amplitude of peak A3 decreases [Fig. 4(*b*) ▸], reflecting the dominant contribution of the *p*_*z*_-DOS to the transition according to the spherical tensor analysis [Fig. S6(*a*) (SI)] in agreement with the calculated DOS [Fig. S7(*b*) (SI)] and conclusions from previous studies (Parlebas *et al.*, 1995 ▸). However, the calculated *p*_*z*_-DOS is only slightly larger than the *p*_*x*,*y*_-DOS [Fig. S7(*b*) (SI)] giving less than 10% amplitude variation with respect to the maximum in the theoretical spectra [Fig. 4(*b*) ▸]. The fitted theoretical evolution of the peak A3 amplitude shows a non-monotonic behaviour indicating the overlapping contributions of a dipolar and quadrupolar transitions. The calculation of the quadrupolar cross-section indicates a contribution in the region of peak A3, which reaches its maximum for θ = 0°[90°] [Fig. 3(*c*) ▸], in agreement with the fitted behaviour which shows that peak A3 originates from the superposition of a dipolar and quadrupolar transition. A quadrupolar evolution with maximum cross-section at θ = 0[90]° is only compatible with the presence of *d*_*xz*_, *d*_*yz*_ states according to the spherical tensor analysis [Fig. S6(*b*) (SI)]. It corresponds to the largest *d*-DOS found in the region of peak A3 [Fig. S7*c*) (SI)]. The quadrupolar contributions of peaks A1 and A3 found in this work are in agreement with temperature-dependent XAS studies in which only quadrupolar transitions undergo a change in cross-section at the metal *K*-edge (Durmeyer *et al.*, 2010 ▸; Brouder *et al.*, 2010 ▸).

At the peak B, the *p*_*z*_-DOS dominates over *p*_*x*,*y*_ [Fig. S7(*b*) (SI)] while all *d*-states have similar contributions except *d*_*xy*_ [Fig. S7(c) SI)]. Experimentally, peak B shows a monotonic decrease in amplitude with θ both experimentally and theoretically [Fig. 4 ▸(*c*)] which is compatible with the dominant *p*_*z*_-DOS identified in our calculations [Fig. S7(*b*) (SI)] and the spherical tensor analysis which predicts a monotonic decay when *p*_*z*_-DOS is dominant [Fig. S6(*a*) (SI)]. Peaks A3 and B are also observed by bremsstrahlung isochromat spectroscopy (BIS) which shows that they originate from essentially empty *p*-DOS which is due to the strong 3*d*–4*p* hybridization for peak A3 and the 4*p*-DOS for peak B (Beaurepaire *et al.*, 1993 ▸), in agreement with our results. The absence of peak A1 in BIS is due to the absence of core-hole effects (Hüfner, 2013 ▸).

Hence, using a combination of *ab initio* FDM calculations and spherical tensor analysis with our experimental LD, the assignment of the dominant final states contributing to the pre-edge transitions at the Ti *K*-edge of r-TiO_2_ is established. The assignments are given in Table 1 ▸.

## 4. Discussion   

### 4.1. Spatial extension of the final states in the pre-edge   

**Figure 5:**
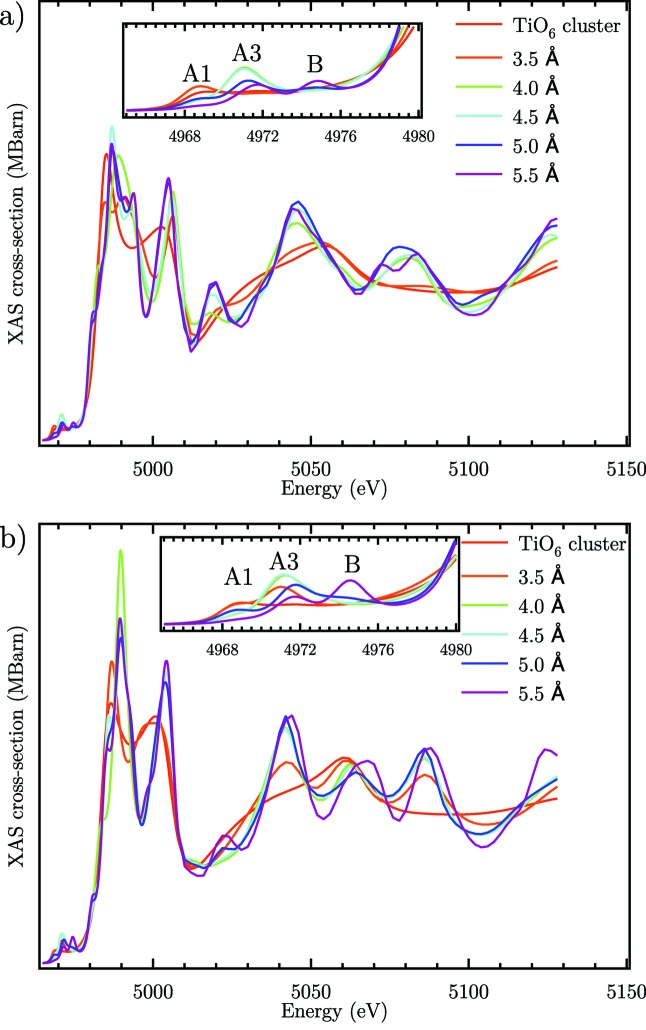
Influence of cluster size on the calculated XAS cross-section (sum of dipole and quadrupole components) (*a*) for θ = 90° and (*b*) for θ = 0°.

The spatial extension of the final states involved in the pre-edge transitions can be approached from the calculations of XAS for various cluster sizes involving more and more distant coordination shells around the absorbing titanium atom. Fig. 5 ▸ shows calculated spectra for increasing cluster sizes from the smallest TiO_6_ octahedron up to a cluster with 5.5 Å radius involving the second nearest-neighbour shell of Ti atoms. Peak A1 can be reproduced with a simple TiO_6_ octahedron in agreement with its quadrupolar nature involving 3*d* final states centred on the absorbing Ti atom (red curve). Peaks A3 and B are absent for the TiO_6_ octahedron showing that they involve final states which are delocalized away from the central TiO_6_ octahedron. The former appears when the nearest coordinating shell of TiO_6_ octahedra is included for a cluster size of 4 Å (green curve) while the latter involves the next nearest neighbour shell which appears for a cluster size of 5.5 Å (purple curve). These results shine a light on the sensitivity of pre-edge peaks to different length scales around the atoms absorbing the radiation. It is clear that the *p*–*d* hybridization occurring in the final states of peak A3 and the pure *p*-DOS component in peak B contribute to their increased sensitivity to coordinating shells. These results are similar to the results obtained on a-TiO_2_ for the peaks A3 and B (Rossi *et al.*, 2019 ▸).

### 4.2. Appearance of transitions to pentacoordinated titanium atoms in the pre-edge   

A remarkable difference between the XAS of a-TiO_2_ and r-TiO_2_ is the absence of a peak A2 in the latter. Previous assignments of this peak in a-TiO_2_ include undercoordinated titanium atoms due to oxygen vacancies (Farges *et al.*, 1997 ▸; Luca *et al.*, 1998 ▸; Luca, 2009 ▸) and structural disorder (Zhang *et al.*, 2008 ▸; Triana *et al.*, 2016 ▸). At high defect concentrations, both phases converge to an amorphous phase where the A2 peak becomes dominant. EXAFS studies have shown that r-TiO_2_ retains a crystalline structure when nanoparticle materials are synthesized by sol-gel (Manzini *et al.*, 1995 ▸) or calcined at different temperatures (Luca, 2009 ▸), which may explain the absence of peak A2. Wu and co-workers have reported changes in the pre-edge of r-TiO_2_ at the Ti *K*-edge from bulk to nanosized materials with a red shift of peak A3 and a red shift and decrease of the amplitude of peak B, while peak A1 is unaffected (Wu *et al.*, 2002 ▸). The most pronounced differences are located in the XANES. Lemercier and co-workers have shown that, under UV irradiation, peak A3 undergoes a red shift which is compatible with the formation of Ti^3+^ centres at the surface of r-TiO_2_ (LeMercier *et al.*, 1995 ▸). Recently, a combined experimental and theoretical study at the Ti *L*_2, 3_-edge of r-TiO_2_ nanoparticles with oxygen vacancies has shown blue-shifted peak replicas from the *t*_2*g*_ and *e*_*g*_ derived final states which are due to 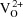 vacancies (Vásquez *et al.*, 2016 ▸). Similar peaks are observed in a-TiO_2_ nanoparticles at the same edge (Thakur *et al.*, 2011 ▸; Krüger *et al.*, 2017 ▸). It is thus surprising that defect-related peaks have been observed in only a few studies at the Ti *K*-edge of r-TiO_2_ while several studies at the O *K*-edge and Ti *L*_2,3_-edge report well defined resonances due to oxygen vacancies (Thomas *et al.*, 2007 ▸; Thakur *et al.*, 2011 ▸; Chen *et al.*, 2015 ▸; Tian *et al.*, 2015 ▸; Vásquez *et al.*, 2016 ▸). They often appear as extra peaks on the high-energy side of the bulk resonances. These resonances become more pronounced with surface-sensitive techniques such as electron energy-loss spectroscopy (EELS) (Göpel *et al.*, 1984 ▸; Eriksen & Egdell, 1987 ▸; Henderson *et al.*, 2003 ▸), which shows the importance of polaron states in the trapping of charges at oxygen vacancies (Eriksen & Egdell, 1987 ▸). These results support our approach of using a relaxed lattice structure around 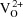 oxygen vacancies (O_vac_) to find the expected pre-edge peaks due to pentacoordinated titanium atoms at the Ti *K*-edge.

**Figure 6:**
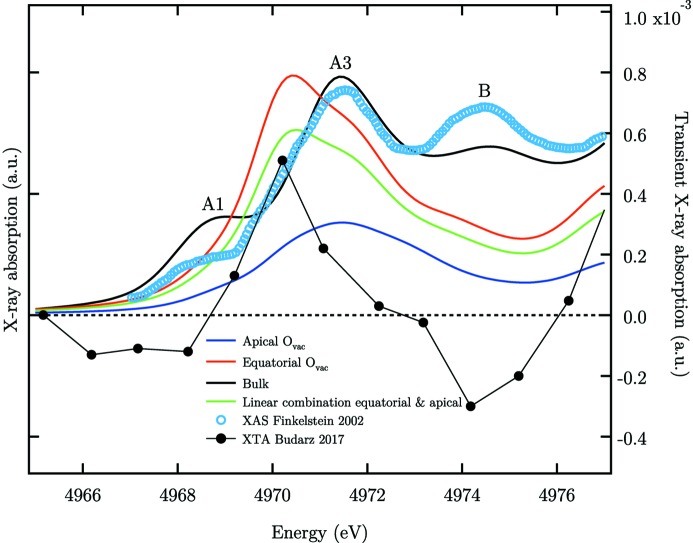
Evolution of the pre-edge of r-TiO_2_ upon formation of 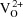 at the Ti *K*-edge. The spectra are calculated for a Ti atom in the bulk structure supercell (black curve), with 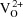 at the apical (blue curve) or the equatorial position (red curve). The calculations are without polarization of the X-ray field (angular average). A linear combination of the apical and equatorial O_vac_ spectra gives a spectrum representative of the titanium atoms surrounding the O_vac_ (green curve). Experimental data from r-TiO_2_ nanoparticles are reproduced from Finkelstein *et al.* (2002 ▸) (blue circles). The spectral trace 100 ps after the excitation of r-TiO_2_ (black line with circle markers) is reproduced from Budarz *et al.* (2017 ▸). The horizontal dashed line represents the zero signal level of the X-ray absorption transient.

A qualitative simulation of the Ti *K*-edge XAS of undercoordinated Ti atoms with O_vac_ in r-TiO_2_ can help in understanding the expected changes from the bulk structure. For these simulations with the full potential FDM, we have used the relaxed local structure from density functional theory calculations with hybrid functionals for the exchange and the correlation terms reported in an earlier work (Janotti *et al.*, 2010 ▸). A bulk 4 × 4 × 4 supercell of r-TiO_2_ is considered first, which contains 739 atoms. A doubly ionized oxygen vacancy (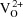) close to the centre of the supercell is simulated by removing an oxygen atom and displacing the nearest Ti atoms by the bond distances reported by Janotti *et al.* (2010 ▸) along the broken Ti—O bonds due to the O_vac_. The XAS are calculated separately at the Ti *K*-edge of the atoms having the O_vac_ in equatorial or in apical position. The results are depicted in Fig. 6 ▸ (blue and red thick lines). Similarly to our previous results on a-TiO_2_ (Rossi *et al.*, 2019 ▸), we find that, for the equatorial O_vac_, peak A1 is absent while a peak appears on the low-energy side of peak A3 (red curve). The XAS of the apical O_vac_ shows mainly one peak centred at the position of peak A3. A similar trend has been previously obtained by others with a smaller cluster size in the multiple scattering approach (Jeanne-Rose *et al.*, 1997 ▸). This additional peak is consistent with DOS appearing at the Ti *L*_2,3_-edge upon formation of oxygen vacancies on the blue side of *t*_2*g*_ and *e*_*g*_-DOS (Vásquez *et al.*, 2016 ▸). These results show that a defect-related resonance due to pentacoordinated Ti atoms may be expected on the low-energy side of peak A3, where the peak A2 is present in a-TiO_2_, which originates from the relaxed projected DOS of Ti atoms in the vicinity of an O_vac_. We noticed that some experimental studies at the Ti *K*-edge of r-TiO_2_ seem to show a peak A3 asymmetry on the red side, suggesting the presence of peak A2 (Manzini *et al.*, 1995 ▸; Finkelstein *et al.*, 2002 ▸). The spectrum measured by Finkelstein and co-workers is shown in Fig. 6 ▸ (blue circles) in which the shoulder at ∼4958 eV is in good agreement with the position of the maximum in our computed spectra with O_vac_. We suspect that peak A2 can only be observed with a substantial amount of 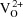 and a monochromator with high energy resolution. The low amount of such vacancies in our crystalline r-TiO_2_ (001) thin films partially explains the absence of peak A2 since the other possible contribution to the formation of this peak is a quadrupolar transition from the *d*-DOS which is discussed in Section 4.3.

Picosecond XAS of photoexcited TiO_2_ nanoparticles shows an enhancement of the spectral signature of trapped charges around metal centres at defects (Rittmann-Frank *et al.*, 2014 ▸). Our picosecond Ti *K*-edge study of r-TiO_2_ nanoparticles (Budarz *et al.*, 2017 ▸) shows the reduction of pentacoordinated Ti centres in the defect-rich shell of the nanoparticles. The transient spectrum at 100 ps after excitation shows an increase in amplitude on the red side of peak A3 and a decrease in peak B amplitude (black line with circle markers in Fig. 6 ▸). To model the XAS of Ti^3+^ centres with a trapped electron without the polarization information, we have taken a linear combination of the calculated apical and equatorial O_vac_ XAS (one-third of the first and two-thirds of the second in agreement with the relative weight of these Ti atoms around the vacancy) which is shown as a green curve in Fig. 6 ▸. A net positive signal appears on the red side of peak A3 and a decrease at peak A1 and B with respect to the bulk spectrum (black curve) in agreement with the transient spectrum (Budarz *et al.*, 2017 ▸). The good qualitative agreement between our simulations and picosecond XAS is evidence of the presence of peak A2 buried under the red side of peak A3 in the XAS of r-TiO_2_ with oxygen vacancies such as in nanoparticles.

We have recently shown that peak A2 is not only due to oxygen vacancies but can also originate from an intrinsic quadrupolar transition in a-TiO_2_ which becomes dominant in single crystals (Rossi *et al.*, 2019 ▸). We investigate the presence of such transition in the pre-edge of r-TiO_2_.

### 4.3. Absence of a quadrupolar A2 peak in the pre-edge of r-TiO_2_ single crystal   

Just as for a-TiO_2_, in r-TiO_2_ the calculated quadrupolar cross-section at peak A3 has a doublet structure which becomes evident at θ ≃ 45° [thin lines in Fig. 3(*c*) ▸]. At θ = 0[90]° the quadrupolar cross-section matches the position of the peak A3 maximum while at θ = 45° the peak is clearly red-shifted with a maximum around 4971 eV. However, this peak is very broad and spans from 4970 to 4973 eV, which prevents formation of a well defined feature at this angle. Additionally, the amplitude of the quadrupolar cross-section near peak A3 is only ∼7% of the total peak amplitude while in a-TiO_2_ the quadrupolar cross-section at peak A2 can be as large as ∼30% (Rossi *et al.*, 2019 ▸). Hence, while *d*-DOS is present in the region of the expected quadrupolar peak A2, the absence of such intrinsic transition in r-TiO_2_ is essentially due to the weak quadrupolar contribution in this region of the spectrum. It originates from the centrosymmetric local point group of rutile (*D*_2*h*_) which generates strict quadrupolar transitions approximately 100 times weaker than dipolar ones (de Groot, 1994 ▸; Yamamoto, 2008 ▸).

Recent calculations dealing explicitly with the core-hole interaction in the Bethe–Salpeter equation have shown that an intrinsic electronic transition on the low-energy side of peak A3 is present in both a-TiO_2_ and r-TiO_2_ (Vorwerk *et al.*, 2017 ▸). The degree of on-site *p*–*d* orbital hybridization is the key parameter for peak A2 to appear as well as the amount of *p*-DOS. In r-TiO_2_, on-site *p*–*d* orbital hybridization is the limiting factor due to the centrosymmetric point group *D*_2*h*_. Hence, we exclude that a quadrupolar transition results in the apparent asymmetry on the low energy side of peak A3 reported in previous studies of nanoparticles since this transition is expected to be too weak to be observed (LeMercier *et al.*, 1995 ▸; Wu *et al.*, 2002 ▸). It is likely due to a transition to pentacoordinated Ti atoms as discussed in Section 4.2. Parlebas and co-workers have reported that a peak A2 is required to fit the experimental XAS in total electron yield of r-TiO_2_ (001) single crystal (Parlebas, 1993 ▸; Parlebas *et al.*, 1995 ▸). However, this shoulder is also present in their BIS data which shows that it does not originate from a blue-shifted quadrupolar transition due to the core-hole effect as would be expected for a quadrupolar peak A2. The presence of a shoulder on the low-energy side of peak A3 in their data is likely due to oxygen vacancies in the single crystal in agreement with our reasoning.

## 5. Conclusion   

In conclusion, we have experimentally investigated in detail the linear dichroism of a rutile TiO_2_ (001) thin film and analyzed it with the support of *FDMNES* *ab initio* calculations and spherical tensor analysis. An unambiguous assignment of the pre-edge transitions is provided which completes the work for the most common rutile and anatase (Rossi *et al.*, 2019 ▸) polymorphs of TiO_2_. The effect of an oxygen vacancy on the XAS of a pentacoordinated Ti atom is investigated for the first time and compared with similar calculations on anatase TiO_2_. The results show that peak A2 is only due to the presence of pentacoordinated titanium atoms at the Ti *K*-edge of r-TiO_2_, in agreement with picosecond XAS (Budarz *et al.*, 2017 ▸). The quadrupolar transition which may be expected from the *d*-DOS in the region of peak A2 cannot be observed because of the limited *p*–*d* hybridization in rutile contrary to anatase TiO_2_ (Rossi *et al.*, 2019 ▸).

## 6. Related literature   

The following references, not cited in the main body of the paper, have been cited in the supporting information: Mo & Ching (1995 ▸); DeVore (1951 ▸); Björck & Andersson (2007 ▸); Als-Nielsen & McMorrow (2011 ▸).

## Supplementary Material

Description of the sample synthesis and characterization, the detailed procedure of the pre-edge fittings, a magnification of the fittings in the pre-edge region, the evolution of the spherical tensor components with incidence angle, the calculated density of states, FDMNES calculations at equivalent sites and the procedure for the crystal symmetrization of the spherical tensors. DOI: 10.1107/S160057752000051X/gb5100sup1.pdf

## Figures and Tables

**Table 1:** Summary of the previous assignments of the pre-edge transitions in the XAS of r-TiO_2_ at the Ti *K*-edge Off-site transitions are shown in bold. E1 stands for dipole transitions and E2 for quadrupolar transitions.

A1	A3	B	Reference
	3*d*(*t*_2*g*_)	3*d*(*e*_*g*_)	(Fischer, 1972 ▸)
E2: 3*d*_*xz*_			(Brouder *et al.*, 1990 ▸)
E2: 3*d*_*xz*_	E1,E2: 3*d*_*xy*,*yz*_	E1,E2: 3*d*_*xy*,*yz*_	(Poumellec *et al.*, 1991 ▸)
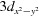	3*d*(*t*_2*g*_)	3*d*(*e*_*g*_)	(Uozumi *et al.*, 1992 ▸)
3*d*–4*p*	3*d*–4*p*	4*p*–4*s*	(Wu *et al.*, 1997 ▸)
Core exciton	3*d*(*t*_2*g*_)	3*d*(*e*_*g*_)	(Beaurepaire *et al.*, 1993 ▸)
E2: 3*d*(*t*_2*g*_)	E1,E2: 3*d*(*e*_*g*_)	**E1: 3*d*–4*p***	(Cabaret *et al.*, 1999 ▸)
	3*d*(*t*_2*g*_)	3*d*(*e*_*g*_)	(Shirley, 2004 ▸)
E2: 3*d*(*t*_2*g*_)			(Le Fèvre *et al.*, 2005 ▸)
E2: 3*d*(*t*_2*g*_)	**E1: *p*_*z*_–3*d*(*t*_2*g*_)**, E2: 3*d*(*e*_*g*_)	**E1: *p*_*z*_–3*d*(*e*_*g*_)**	(Cabaret *et al.*, 2010 ▸) ( 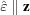 )
E2: 3*d*(*t*_2*g*_)	**E1: *p*_*x*, *y*_–3*d*(*t*_2*g*_)**	**E1: *p*_*x*, *y*_–3*d*(*e*_*g*_)**	(Cabaret *et al.*, 2010 ▸) ( 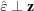 )
E2: 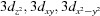 ,E1: 4*p*_*x*,*y*_	**E1: 4*p*_*z*_,3*d*_*xz*,*yz*_**	**E1: 4*p*_*z*_**	(This work)

**Table 2:** Angular evolution of the dominant term in the dipole and quadrupole cross-section with Euler angle θ as defined in the main text for a given final state in the monoelectronic approximation

Final state	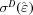 or 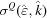 θ-dependence
*p*_*x*_,*p*_*y*_	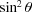
*p*_*z*_	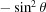
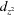	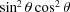
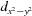	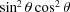
*d*_*xy*_	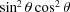
*d*_*xz*_	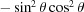
*d*_*yz*_	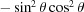

## Footnotes

1 This choice of nomenclature can be found in previous works (Wu *et al.*, 1997 ▸). The usual nomenclature in which the pre-edge peaks are labelled A1, A2 and A3 is misleading since it does not allow a direct comparison with the pre-edge peaks of anatase, another polymorph of TiO_2_. In particular, it was recently shown that the peak A2 in anatase single crystals originates from a quadrupole allowed transition (Rossi *et al.*, 2019 ▸) which is not the case for the usually labelled peak A2 of rutile [see this work and Cabaret *et al.* (2010 ▸)]. The absence of a similar quadrupolar peak A2 in the pre-edge of rutile is detailed in the current work and originates from the centrosymmetry of the TiO_6_ octahedra leading to a weak transition not experimentally observable. The presence of quadrupolar peak A2 in rutile is predicted by recent state-of-the-art calculations on the low-energy side of peak A3 (Vorwerk *et al.*, 2017 ▸). Hence, the choice of nomenclature in this work allows a direct comparison between the pre-edge of anatase and rutile in which the same labels correspond to similar final states (based on the nature of the orbitals involved and spatial extension).

2 For a spectrum measured well above the absorption edge, the atomic background absorption converges to the same amplitude for any incident polarization and can also be used in principle to renormalize the spectra.
