# Supplementary material for: X-ray absorption linear dichroism at the Ti K-edge of rutile (001) TiO2 single crystal
Source: J Synchrotron Radiat. 2020 Feb 14;27(Pt 2):425–35. doi: 10.1107/S160057752000051X (PMC7064109; doi:10.1107/S160057752000051X)
Supplement: Supplementary file 1 [file s-27-00425-sup1.pdf]

X-ray absorption linear dichroism at the Ti K-edge of rutile (001) single crystal

T. C. Rossi[1], D. Grolimund[2], O. Cannelli[1], G. F. Mancini[1], C. Bacellar[1], D. Kinschel[1], J. R. Rouxel[1], N. Ohannessian[3], D. Pergolesi[3,4], and M. Chergui[1]

[1]Laboratory of Ultrafast Spectroscopy, Ecole Polytechnique Fédérale de Lausanne SB-ISIC-LSU, and Lausanne Centre for Ultrafast Science (LACUS), Station 6, Lausanne, CH-1015, Switzerland

[2]Laboratory for Femtochemistry - MicroXAS beamline project, Paul Scherrer Institute, Villigen, CH-5232, Switzerland

[3]Laboratory for Multiscale Materials Experiments, Paul Scherrer Institute, Villigen, CH-5232, Switzerland

[4]Electrochemistry Laboratory, Paul Scherrer Institute, Villigen, CH-5232, Switzerland

November 22, 2019

Table of contents

1 Sample synthesis and characterization 3

1.1 Synthesis . . . 3

1.2 X-ray diffraction and rocking curve . . . 3

1.3 Ellipsometry . . . 3

1.4 X-ray reflectometry . . . 5

2 Fitting of the pre-edge peaks 6

3 Linear dichroism in the pre-edge 8

4 Evolution of the spherical tensor components with incidence angle 9

5 Calculated density of states 10

6 Calculation per site 11

7 Crystal-symmetrization of the spherical tensors 12

7.1 Derivation of the site-symmetrized spherical tensors of rutile in the site frame and in the crystal frame . . . 12

7.2 Derivation of the crystal-symmetrized spherical tensors of rutile in the crystal frame 14

7.3 Expressions of the spherical tensors . . . 15

7.4 Expression of the dipole and quadrupole cross-section assuming a final state . . . 17

7.4.1 Final state is p_z . . . 17

7.4.2 Final state is p_x or p_y . . . 17

7.4.3 Final state is d_{z^2} . . . 17

7.4.4 Final state is $d_{x^2-y^2}$ . . . 17

7.4.5 Final state is d_{xy} . . . 17

7.4.6 Final state is d_{xz},d_{yz} . . . 17

1 Sample synthesis and characterization

1.1 Synthesis

(001)-oriented TiO_2 rutile thin films were prepared by Pulsed Laser Deposition. The vacuum chamber was evacuated to a base pressure of 10^{-5} Pa. The samples were grown by ablating a commercially available TiO_2 target with a KrF excimer laser (Lambda Physik LPX 300, 25 ns pulses, $\lambda = 248$ nm). The energy density was set at 3.0 J/cm^2 with a spot size of about 1.5 mm^2. The films were grown under an O_2 partial pressure of 10 Pa. The target-substrate distance was fixed at 40 mm. The rutile films were grown on $10 \times 10 \times 0.5$ mm double side polished, $(10\bar{1}0)$-oriented sapphire substrates (CrysTec GmBH). The substrate temperature was set at 670°C read out using a pyrometer directed onto a spot of Pt paste painted on the sample holder near the substrate edge. The thermal contact between substrate and heating stage was provided by Pt paste.

1.2 X-ray diffraction and rocking curve

The crystalline structure of the samples was determined by X-ray Diffraction (PANalytical X'pert Pro MPD with Cu $K_{\alpha 1}$ radiation at 1.540 Å). The X-ray diffraction pattern of the TiO_2 thin film is shown in Figure 1a which confirms the (001)-orientation of the film. The diffraction pattern shows two peaks at 62.9° and 68.5° corresponding to the (002) diffraction of r-TiO_2 and the $(10\bar{1}0)$ diffraction of the M-sapphire substrate, respectively. Figure 1b shows a rocking curve measured at the rutile (002) diffraction peak. The full width at half maximum is 0.48° associated to a mean crystallite size of about 3.4 nm. The in-plane lattice mismatch between the film ($a_{rutile} = 4.594$ Å) and the substrate ($a_{sapphire} = 4.785$ Å) is about -4.0 %. Despite this considerable value, the M-plane sapphire represents a suitable platform for an epitaxial growth of (001)-oriented r-TiO_2.

1.3 Ellipsometry

The ellipsometry measurements were carried out at room temperature with a SE-2000 spectroscopic ellipsometer from Semilab and microspot optics in rotating attenuator mode. Prior to each measurement, the vertical position of the sample was optimized for maximal reflection intensity. The absorption coefficient was measured by spectroscopic ellipsometry on the thin film of r-TiO_2

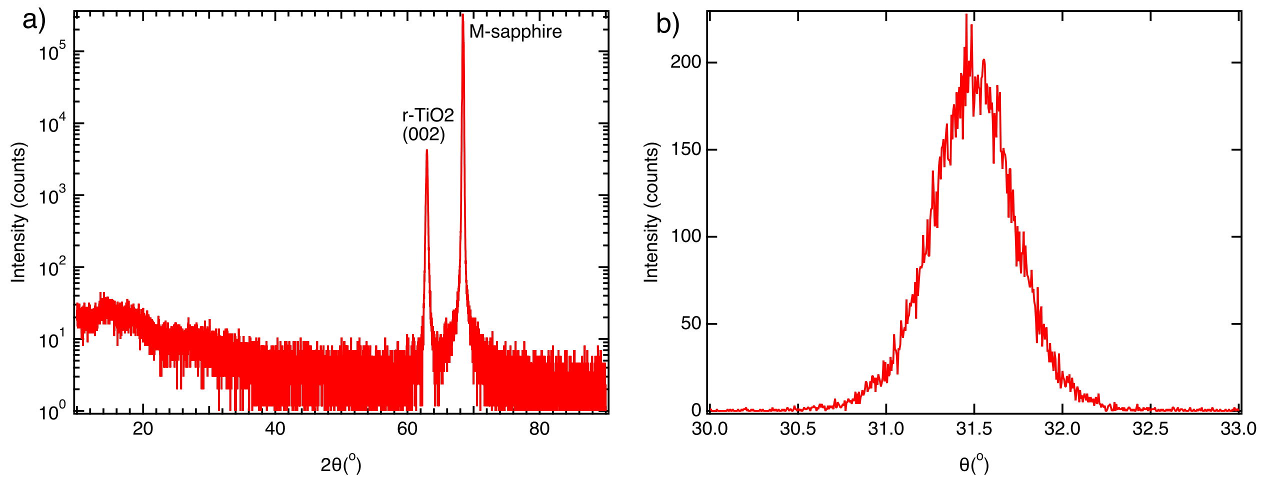

Figure 1: a) X-ray diffraction pattern of the r-TiO_2 (001) thin film. b) Rocking curve around the (002) diffraction peak.

(001) on sapphire substrate. To ensure that the sample remains homogeneous in optical properties and in thickness, we have measured the absorption coefficient spectrum at 9 different points at the sample surface which are arranged in a 3×3 square grid with 1 mm spacing between the points. The spectra are given in Figure 2a. The region of the optical gap with a maximum at 4 eV is identical at every site showing that it is homogeneous across the surface. The position of this maximum absorption coefficient is in excellent agreement with previously published data on r-TiO_2 with the (001) orientation showing the bulk-like optical properties of the crystalline thin film [1]. The oscillations below the gap in the near-IR and Visible are due to Fabry-Perot interferences which depend on the thickness of the r-TiO_2 layer. We have estimated fluctuations in sample thickness from the energy shift of the peak at $\sim$2.3 eV. The position of this peak maximum spans an energy range of 0.1 eV which, with the refractive index of r-TiO_2 at this energy of 2.67 [2] and the average thickness provided by X-ray reflectometry of 45.9 nm (see section 1.4), the variation in thickness of the material derived from Fabry-Perot formula,

$$\Delta L = \frac{\Delta E}{E}\frac{L}{2n} \tag{1}$$

with L the sample thickness, E the energy of the resonance and n the refractive index, we estimate a thickness fluctuation of 3.7 Å which is less than 1% of the total film thickness.

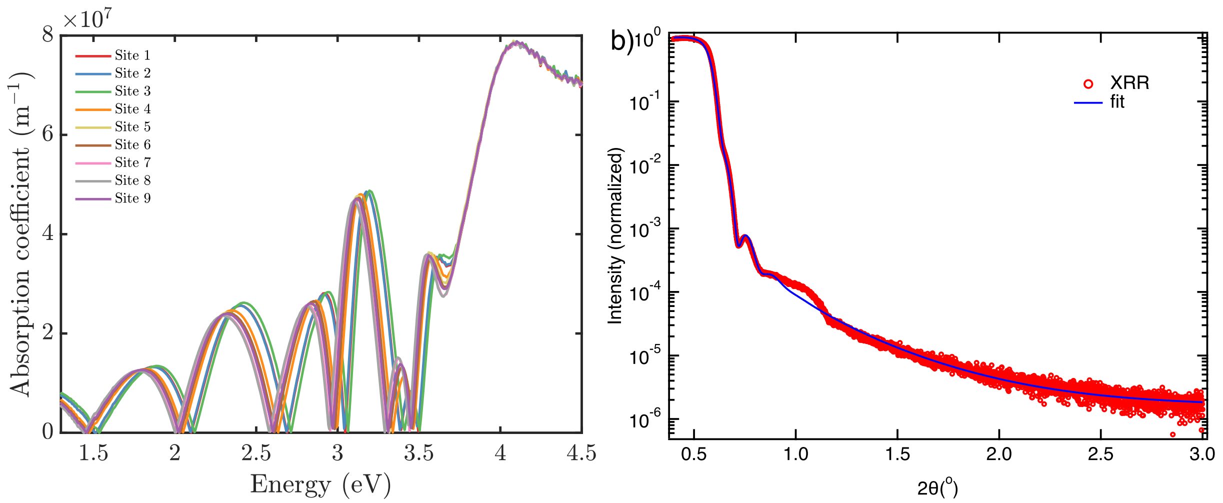

Figure 2: a) Absorption coefficient spectra measured by ellipsometry at 9 different points at the r-TiO_2 (001) thin film surface. b) Experimental X-ray reflectometry from r-TiO_2 (001) (red circles) and fitting (blue line).

1.4 X-ray reflectometry

The X-ray reflectometry was measured with a Bruker D8 Advance (Cu $K_{\alpha 1}$ radiation at 1.540 Å). A vertical slit of 0.1 mm is used to limit the vertical extension of the X-rays at grazing incidence. Soler slits are used to select the diffracted beam. The experimental X-ray reflectometry is shown in Figure 2b. The experimental data was fitted with the open source `GenX` software [3]. Effect of incident intensity and background intensity variation were included. A bulk alumina substrate was considered with a bulk r-TiO_2 layer on top ($a = b = 4.593$ Å, $c = 2.959$ Å, $\alpha = \beta = \gamma = 90°$). Surface roughness was included for both the r-TiO_2 layer and the alumina substrate. The r-TiO_2 layer thickness is fitted to 45.9 ± 0.5 nm.

2 Fitting of the pre-edge peaks

The pre-edge in the experimental data was fitted in `Matlab` with a set of four gaussians while the pre-edge from the theory was fitted with a set of four pseudo-Voigt functions. Overlap between the spectra and the fits are shown in Figure 3. Fittings of experimental spectra at a given incidence angle are shown in Figure 4. The X-ray absorption spectrum (XAS) amplitude in the experiment and in the theory do not match because the experiment deals with a number of photons detected by an APD in a given emission cone while the calculations provide absorption cross-sections. The detected fluorescence yield per incident photon depends on the linear absorption coefficient μ in a non-linear fashion [4],

$$\frac{I_f}{I_0} = \epsilon \left(\frac{\Delta\Omega}{4\pi} \right) e^{-a\mu_f d'} \frac{\mu}{\mu - a\mu_f} \left[1 - e^{-(\mu - a\mu_f)d'} \right] \tag{2}$$

where ϵ is the probability to emit a X-ray fluorescence photon, $\Delta\Omega$ the solid angle of detection, μ_f the linear absorption coefficient of the fluorescence through a path length d' inside the sample. In the thin film approximation, $(\mu - a\mu_f)d' \to 0$ such that the fluorescence yield becomes,

$$\frac{I_f}{I_0} = \epsilon \left(\frac{\Delta\Omega}{4\pi} \right) (1 - \mu_f a d')\mu d' \tag{3}$$

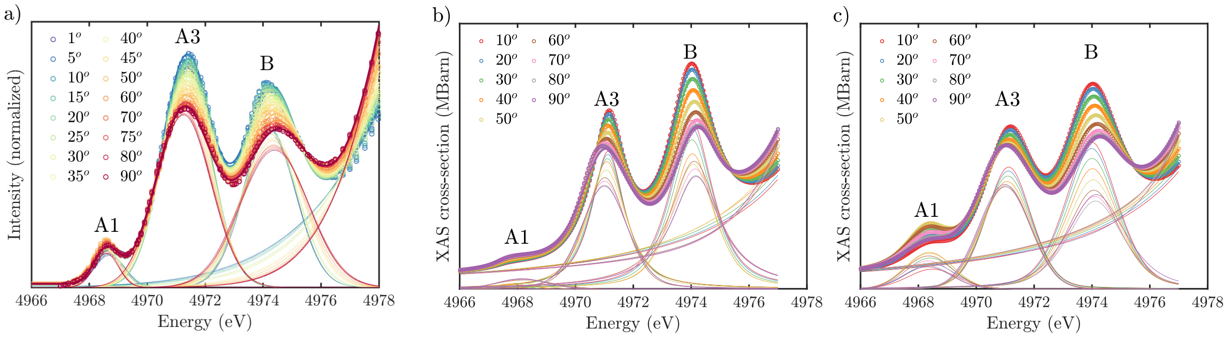

Figure 3: a) Experimental pre-edge of r-TiO_2 [001] (circles) overlapped with fitting curves based on a set of four gaussians (continuous lines). b) Calculated pre-edge of r-TiO_2 from $\theta = 0°$ to $90°$ with dipole matrix elements only (circles). Fitting with a set of four pseudo-Voigt functions (continuous lines). c) Calculated pre-edge of r-TiO_2 with dipole and quadrupole matrix elements (circles). Fitting with a set of four pseudo-Voigt functions (continuous lines).

The normalization at the isosbestic points predicted by the theory accounts for the changes in $\Delta\Omega$ providing spectra proportional to μ. The linear absorption coefficient is linearly related to the

absorption cross-section σ by,

$$\sigma = \frac{\mu}{N} \tag{4}$$

where N is the atomic density. Hence, a multiplicative coefficient needs to be applied to the experimental data after normalization to match the evolution of the theoretical absorption cross-section providing the experimental points depicted in Figure 4 of the main text.

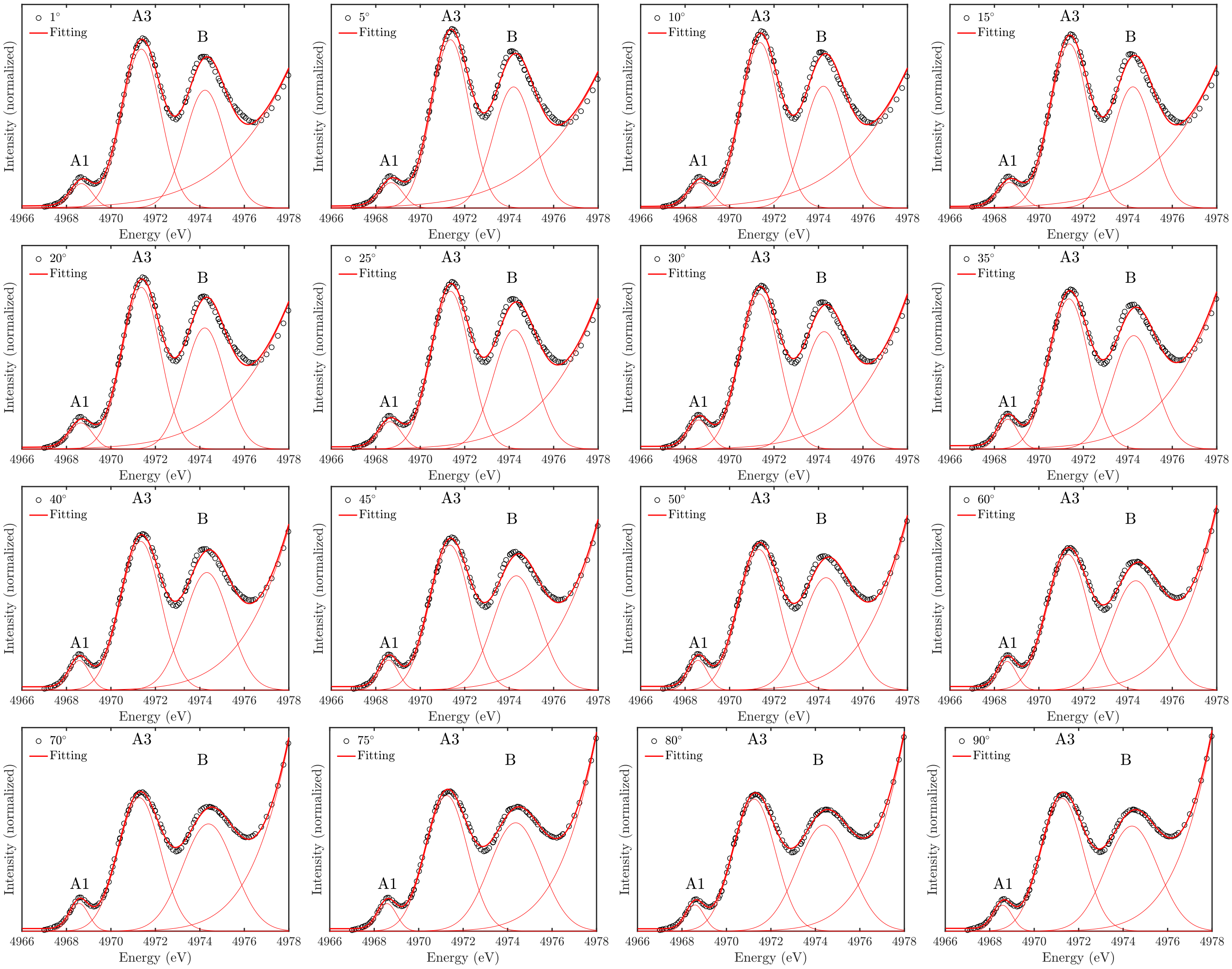

Figure 4: Individual fittings of the experimental r-TiO_2 XAS with incidence angle θ. Experimental data are the black circles. Fitting with a sum of four gaussians is shown with thick red lines. Individual gaussian lineshapes are shown with thin red lines lines.

3 Linear dichroism in the pre-edge

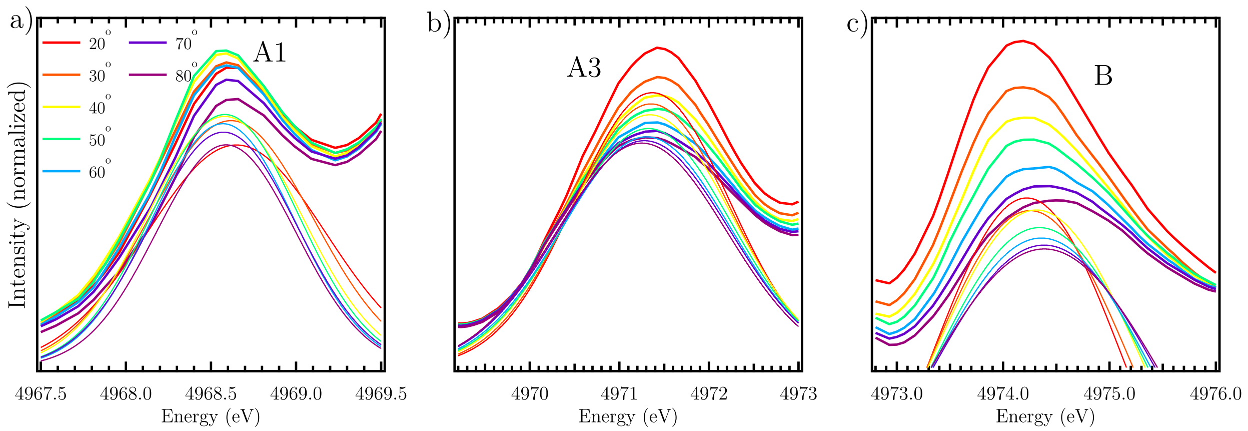

Figure 5: Experimental evolution of the pre-edge peaks of r-TiO_2 (001) with the rotation angle θ (thick lines). Fitting of the experimental with gaussian lineshapes are shown with thin lines with the color corresponding to each incidence angle in the legend. The vertical scale is different in all figures for better visibility between the experimental data and the fits.

4 Evolution of the spherical tensor components with incidence angle

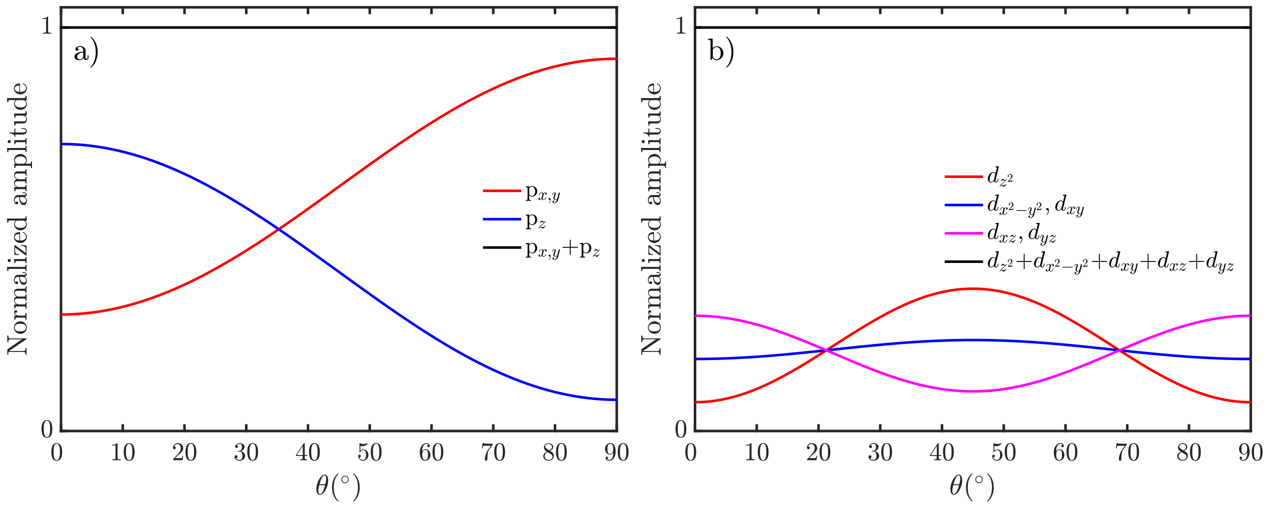

Figure 6: Evolution of the a) dipole and b) quadrupole cross-section predicted by the spherical tensor analysis with incidence angle θ assuming a given orbital in the final state reached by the transition at the Ti K-edge.

5 Calculated density of states

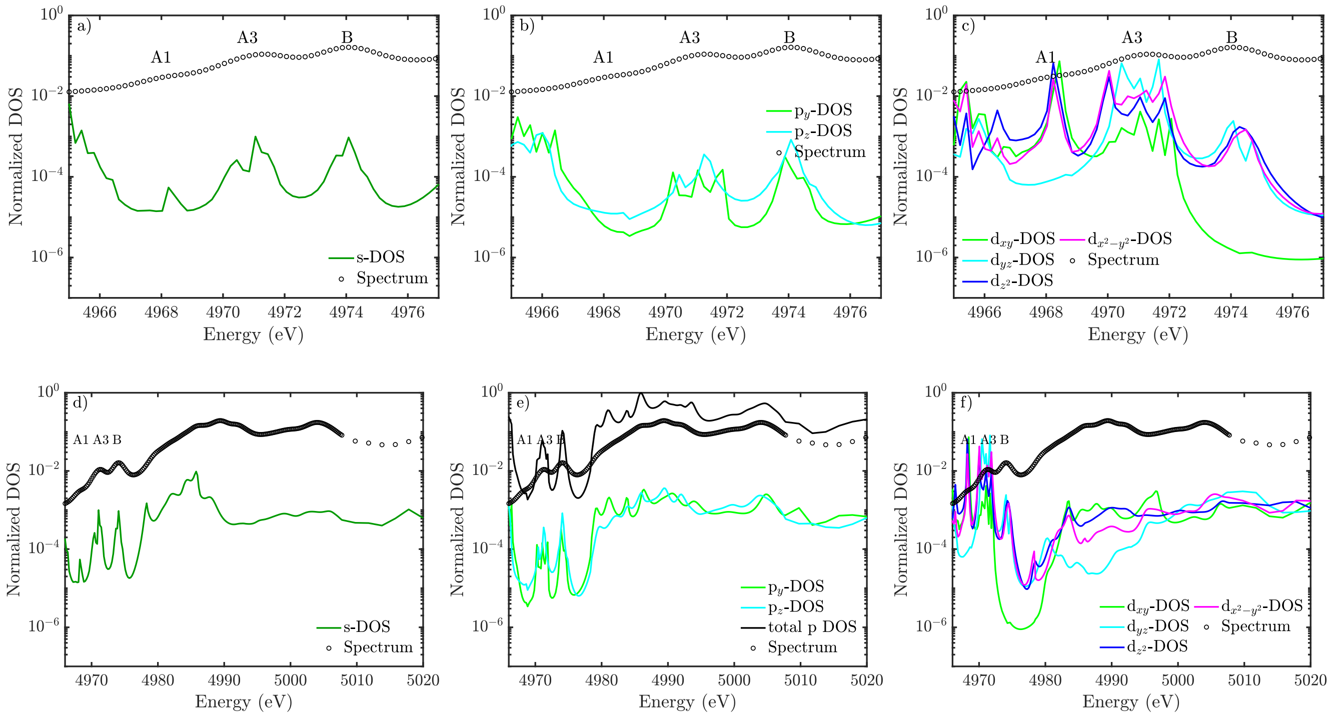

Figure 7: Calculation of a),d) s, b),e) p and c),f) d-DOS in the pre-edge (a-c) and in the XANES (d-f). The DOS of each orbital is normalized to the orbital with the largest DOS among s-, p- and d-orbitals. The similarity between the calculated spectrum and the integrated p-DOS is shown in e).

6 Calculation per site

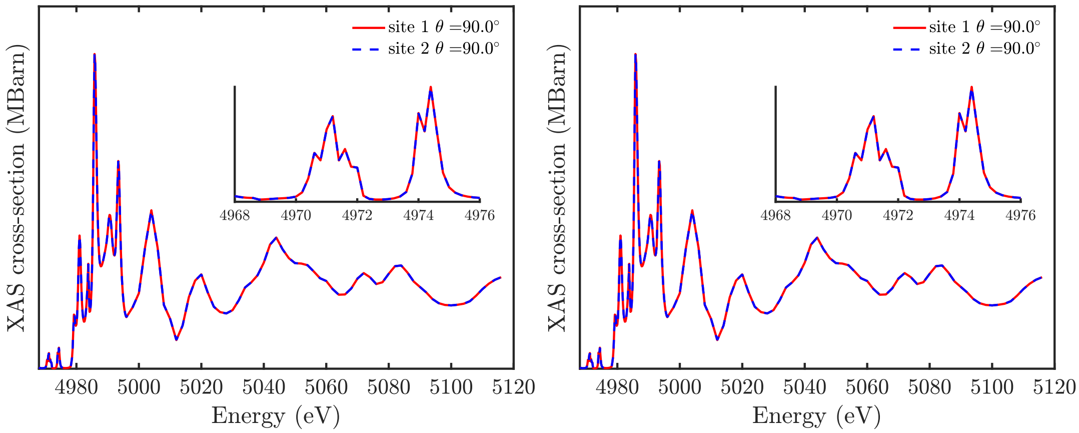

Figure 8: Evolution of the a) dipole and b) quadrupole XAS at the Ti K-edge of r-TiO_2 at $\theta = 90°$ for the two equivalent Ti sites in the unit cell. The spectra are taken for $\phi = 0°$. No convolution is applied to the core-hole lifetime or to the experimental energy resolution.

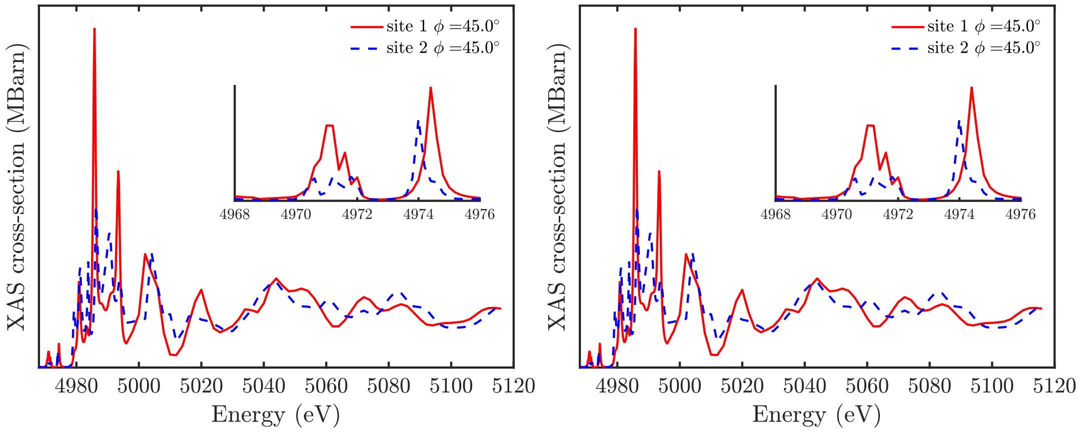

Figure 9: Evolution of the a) dipole and b) quadrupole XAS at the Ti K-edge of r-TiO_2 at $\phi = 45°$ for the two equivalent Ti sites in the unit cell. The spectra are taken for $\theta = 90°$. No convolution is applied to the core-hole lifetime or to the experimental energy resolution.

7 Crystal-symmetrization of the spherical tensors

First, the spherical tensors are symmetrized in the atomic frame and then in the crystal frame taking into account the possible inequivalent sites in the unit cell.

7.1 Derivation of the site-symmetrized spherical tensors of rutile in the site frame and in the crystal frame

The frame set around a Ti site has the $\hat{z}$ axis chosen along the elongated axis of the TiO_6 octahedron and the $\hat{x}$ and $\hat{y}$ axes are chosen along the equatorial Ti–O bonds to form a direct frame. Hence, there is a mismatch between the Euler angles in the site frame and in the crystal frame. The rotation to go from the crystal frame to the site frame is given by the matrix R,

$$R = \begin{pmatrix} \frac{\sqrt{2}}{2} & -\frac{\sqrt{2}}{2} & -\frac{\sqrt{2}}{2} \\ -\frac{\sqrt{2}}{2} & \frac{\sqrt{2}}{2} & -\frac{\sqrt{2}}{2} \\ \frac{\sqrt{2}}{2} & \frac{\sqrt{2}}{2} & 0 \end{pmatrix} \tag{5}$$

We would like to go from the site frame to the crystal frame which is given by the transformation matrix R^{-1} with Euler angles $(-3\pi/4, -\pi/2, 3\pi/4)$ in ZYZ convention. The site-symmetrized spherical tensor $\langle\sigma(l,m)\rangle$ in the site frame is given by:

$$\langle\sigma(l,m)\rangle = \frac{1}{|G'|}\sum_{g}\epsilon(g)\sum_{m'=-l}^{m'=l}\sigma(l,m')D^{l}_{m'm}(g) \tag{6}$$

with $|G'| = 8$ the number of pure rotations and rotoinversions in the site point group, g a rotoinversion in the site point group, and $D^{l}_{m'm}$ a Wigner-D matrix element. The parity factor is $\epsilon(g) = -1$ when the spherical tensor is odd and the symmetry operation g contains the inversion or $\epsilon(g) = 1$ otherwise. The Euler angles associated to the symmetry operations in the point group are, E: $(0,0,0)$, $C_2(x)$: $(0,-\pi,\pi)$, $C_2(y)$: $(0,-\pi,0)$, $C_2(z)$: $(0,0,\pi)$, i: $(0,-\pi,\pi)$, $\sigma(xy)$ $(0,-\pi,0)$, $\sigma(xz)$: $(0,0,\pi)$ and $\sigma(yz)$: $(0,0,0)$. This provides the following symmetrized dipolar spherical tensors in the site frame:

$$\begin{aligned} \langle\sigma^{D}(0,0)\rangle &= \frac{1}{8}\sum_{g}\epsilon(g)\sigma^{D}(0,0)D^{0}_{00}(g) = \sigma^{D}(0,0) \\ \langle\sigma^{D}(2,0)\rangle &= \frac{1}{8}\sum_{g}\sum_{m'=-2}^{m'=2}\epsilon(g)\sigma^{Q}(2,m')D^{2}_{m'0}(g) = \sigma^{D}(2,0) \end{aligned} \tag{7}$$

and the following quadrupolar spherical tensors,

$$
\begin{aligned}
\langle\sigma^Q(0,0)\rangle &= \frac{1}{8}\sum_g \sigma^Q(0,0)D^0_{00}(g) = \sigma^Q(0,0)\\
\langle\sigma^Q(2,0)\rangle &= \frac{1}{8}\sum_g\sum_{m'=-2}^{m'=2} \sigma^Q(2,m')D^2_{m'0}(g) = \sigma^Q(2,0)\\
\langle\sigma^Q(4,0)\rangle &= \frac{1}{8}\sum_g\sum_{m'=-4}^{m'=4} \sigma^Q(4,m')D^4_{m'0}(g) = \sigma^Q(4,0)\\
\langle\sigma^Q(4,4)\rangle &= \frac{1}{8}\sum_g\sum_{m'=-4}^{m'=4} \sigma^Q(4,m')D^4_{m'4}(g) = \Re\sigma^Q(4,4)
\end{aligned} \tag{8}
$$

Now, we can convert these site-symmetrized tensors from the site frame to the crystal frame with the rotation R^{-1} in matrix 5. This is performed with the formula,

$$
\langle\sigma(l,m)\rangle_2 = \sum_{m'=-l}^{m'=l} \langle\sigma(l,m')\rangle_1 \, D^l_{m'm}(g) \tag{9}
$$

where g are the Euler angles associated to the symmetry operation to go from frame 1 to the frame 2. The Euler angles associated to R^{-1} are $(-3\pi/4, -\pi/2, 3\pi/4)$. It provides the transformed site-symmetrized cross-sections for the dipole cross-section,

$$
\begin{aligned}
\langle\sigma^D(0,0)\rangle &= \sigma^D(0,0)\\
\langle\sigma^D(2,0)\rangle &= \sigma^D(2,0)
\end{aligned} \tag{10}
$$

and for the quadrupole cross-section,

$$
\begin{aligned}
\langle\sigma^Q(0,0)\rangle &= \sigma^Q(0,0)\\
\langle\sigma^Q(2,0)\rangle &= -\frac{1}{2}\sigma^Q(2,0)\\
\langle\sigma^Q(4,0)\rangle &= -\frac{1}{4}\sqrt{\frac{35}{2}}\Re\sigma^Q(4,4) + \frac{3}{8}\sigma^Q(4,0)\\
\langle\sigma^Q(4,4)\rangle &= \frac{1}{8}\Re\sigma^Q(4,4) - \frac{1}{8}\sqrt{\frac{35}{2}}\sigma^Q(4,0)
\end{aligned} \tag{11}
$$

where we have used the time-reversal symmetry relation $\sigma(l,m)^* = (-1)^m\sigma(l,-m)$, the fact that $\sigma^Q(4,2)$ and $\sigma^Q(2,2)$ are real so that $\Im\sigma^Q(4,2) = \Im\sigma^Q(2,2) = 0$ and the fact that $\sigma^Q(4,3) = \sigma^Q(4,1) = 0$ [5]. Now that we have the site-symmetrized tensors in the crystal frame, we can calculate the crystal symmetrized tensors which enter equations (3) and (4) in the main text.

7.2 Derivation of the crystal-symmetrized spherical tensors of rutile in the crystal frame

The coset method is a powerful way to calculate the spherical tensors averaged over the crystal from the spherical tensor symmetrized over a single site which has been developed by Brouder and coworkers [6]. The crystal-symmetrized tensor $\langle\sigma(l,m)\rangle_X$ of a given site is obtained from the site-symmetrized tensor $\langle\sigma(l,m)\rangle$ by the operation

$$\langle\sigma(l,m)\rangle_X = \frac{1}{n}\sum_{i=1}^{n}\sum_{m'=-l}^{m'=l} \epsilon(g_i)\,\langle\sigma(l,m)\rangle\, D^l_{m'm}(g_i^{-1}) \tag{12}$$

with n the number of cosets which is given by the ratio between the number of symmetry operations of the space group of rutile ($P_{4_2/mnm}$, group number 136 with choice of origin number 1) and the number of symmetry operations in a reduced space group with periodic translations which leave a given Ti site invariant which are 8 in total: (x,y,z), $(-x,-y,z)$, (y,x,z), $(-y,-x,z)$, $(-x,-y,-z)$, $(x,y,-z)$, $(-y,-x,-z)$ and $(y,x,-z)$, providing 2 cosets (or two equivalent Ti sites) whose representatives are (x,y,z) and $(-y+\frac{1}{2},x+\frac{1}{2},z+\frac{1}{2})$. The symmetry operation which allows going from (x,y,z) to $(-y+\frac{1}{2},x+\frac{1}{2},z+\frac{1}{2})$ is represented by the rotation matrix R (without translations),

$$R = \begin{pmatrix} 0 & -1 & 0 \\ 1 & 0 & 0 \\ 0 & 0 & 1 \end{pmatrix}. \tag{13}$$

The Euler angles associated to R^{-1} required in equation 12 are $(0,0,-\pi/2)$ in ZYZ convention. We can now calculate the crystal-symmetrized tensors in the crystal frame and give an expression with respect to the site-symmetrized tensors in the site frame by taking the average over the equivalent sites. It provides for the dipole cross-section,

$$\begin{aligned} \langle\sigma^D(0,0)\rangle_X &= \langle\sigma^D(0,0)\rangle = \sigma^D(0,0) \\ \langle\sigma^D(2,0)\rangle_X &= \langle\sigma^D(2,0)\rangle = -\frac{1}{2}\sigma^D(2,0) \end{aligned} \tag{14}$$

and for the quadrupole cross-section,

$$\begin{aligned}
\langle\sigma^Q(0,0)\rangle_X &= \langle\sigma^Q(0,0)\rangle = \sigma^Q(0,0) \\
\langle\sigma^Q(2,0)\rangle_X &= \langle\sigma^Q(2,0)\rangle = -\frac{1}{2}\sigma^Q(2,0) \\
\langle\sigma^Q(4,0)\rangle_X &= \langle\sigma^Q(4,0)\rangle = -\frac{1}{4}\sqrt{\frac{35}{2}}\sigma^Q(4,4) + \frac{3}{8}\sigma^Q(4,0) \\
\langle\sigma^Q(4,4)\rangle_X &= \langle\sigma^Q(4,4)\rangle = \frac{1}{8}\Re\sigma^Q(4,4) - \frac{1}{8}\sqrt{\frac{35}{2}}\sigma^Q(4,0)
\end{aligned} \tag{15}$$

Introducing the values of the spherical tensors in Tables 1 and 2 we can now calculate the expected expression of the dipole and quadrupole cross-section assuming a given final state represented by a single orbital in the monoelectronic approximation. This provides the angular dependencies for the dipole and quadrupole cross-section given in Table 2 of the main text.

7.3 Expressions of the spherical tensors

The dipole spherical tensors can be reduced to:

$$\sigma^D(l,m) = \sum_{\mu\mu'}(1\mu 1\mu'|lm)\sigma^D(1,\mu,1,\mu') \tag{16}$$

with $(1\mu 1\mu'|lm)$ the corresponding Clebsch-Gordan coefficient ($-l \leq \mu \leq l$, $-l \leq \mu' \leq l$) and

$$\begin{aligned}
\sigma^D(1,\mu,1,\mu') = -\pi\alpha\hbar\omega(4\pi/3)^2\sqrt{3}\sum_{if}\langle i|\, rY_1^{\mu}(\hat{r})\,|f\rangle\,\langle f|\, rY_1^{\mu'}(\hat{r})\,|i\rangle \\
\times\delta(E_f - E_i - \hbar\omega)
\end{aligned} \tag{17}$$

with $|i\rangle$ the initial state of the transition (1s orbital in this work) and $|f\rangle$ the final state. Y_l^m is the spherical harmonic with angular moment number l and magnetic quantum number m. The quadrupole spherical tensors can be reduced to:

$$\sigma^Q(l,m) = \sum_{\nu\nu'}(2\nu 2\nu'|lm)\sigma^Q(2,\nu,2,\nu') \tag{18}$$

with

$$\begin{aligned}
\sigma^Q(2,\nu,2,\nu') = 3\pi\alpha\hbar\omega k^2/(20\sqrt{5})\sum_{if}\langle i|\, r^2Y_2^{\nu}(\hat{r})\,|f\rangle\,\langle f|\, r^2Y_2^{\nu'}(\hat{r})\,|i\rangle \\
\times\delta(E_f - E_i - \hbar\omega)
\end{aligned} \tag{19}$$

In these relations, the spherical integral over r or r^2 should not be forgotten and we do not compute it explicitly by introducing the parameters ξ_f^2.

**final state**	$\sigma^D(0,0)$	$\sigma^D(2,0)$
p_x	$2/\sqrt{5}$	$-2/\sqrt{14}$
p_y	$2/\sqrt{5}$	$-2/\sqrt{14}$
p_z	$2/\sqrt{5}$	$2/\sqrt{14}$

Table 1: Table of dipolar spherical tensors based on the reached final state. Each element of the table must be multiplied by $\alpha\hbar\omega\delta(E_f - E_i - \hbar\omega)\xi_f^2$ where f is the final state reached by the transition which represents the integration of the radial parts of the wavefunctions.

**final state**	$\sigma^Q(0,0)$	$\sigma^Q(2,0)$	$\sigma^Q(2,2)$	$\sigma^Q(4,0)$	$\sigma^Q(4,2)$	$\sigma^{Qr}(4,4)$	$\sigma^{Qr}(4,-4)$
d_{z^2}	$2/\sqrt{5}$	$-4/\sqrt{14}$	0	$12/\sqrt{70}$	0	0	0
$d_{x^2-y^2}$	$2/\sqrt{5}$	$4/\sqrt{14}$	0	$2/\sqrt{70}$	0	1	1
d_{xy}	$2/\sqrt{5}$	$4/\sqrt{14}$	0	$2/\sqrt{70}$	0	-1	-1
d_{xz}	$2/\sqrt{5}$	$-2/\sqrt{14}$	$-\sqrt{3/7}$	$-8/\sqrt{70}$	$2/\sqrt{7}$	0	0
d_{yz}	$2/\sqrt{5}$	$-2/\sqrt{14}$	$\sqrt{3/7}$	$-8/\sqrt{70}$	$-2\sqrt{7}$	0	0

Table 2: Table of quadrupolar spherical tensors based on the reached final state. Each element of the table must be multiplied by $\alpha\hbar\omega k^2\delta(E_f - E_i - \hbar\omega)\xi_f^2$ where f is the final state reached by the transition which represents the integration of the radial parts of the wavefunctions.

7.4 Expression of the dipole and quadrupole cross-section assuming a final state

7.4.1 Final state is $\mathbf{p}_z$

$$\sigma^D(\hat{\epsilon}) \propto \alpha\hbar\omega \left(\frac{2}{\sqrt{5}} - \frac{1}{\sqrt{7}}(3\sin^2\theta - 1)\right) \xi_{z^2}^2 \delta\left(E_f - E_i - \hbar\omega\right) \tag{20}$$

7.4.2 Final state is $\mathbf{p}_x$ or $\mathbf{p}_y$

$$\sigma^D(\hat{\epsilon}) \propto \alpha\hbar\omega \left(\frac{2}{\sqrt{5}} + \frac{1}{\sqrt{7}}(3\sin^2\theta - 1)\right) \xi_{x,y}^2 \delta(E_f - E_i - \hbar\omega) \tag{21}$$

7.4.3 Final state is d_{z^2}

$$\sigma^Q(\hat{\epsilon}, \hat{k}) \propto \alpha\hbar\omega k^2 \left(\frac{2}{\sqrt{5}} + \frac{6}{7\sqrt{5}}(35\sin^2\theta\cos^2\theta - 4)\right) \xi_{z^2}\delta(E_f - E_i - \hbar\omega) \tag{22}$$

7.4.4 Final state is $d_{x^2-y^2}$

$$\sigma^Q(\hat{\epsilon}, \hat{k}) \propto \alpha\hbar\omega k^2 \left(\frac{2}{\sqrt{5}} + \frac{1}{7\sqrt{5}}(35\sin^2\theta\cos^2\theta - 4) - \sqrt{5}\sin^2\theta\cos^2\theta\sin 4\phi\right) \xi_{x^2-y^2}\delta(E_f - E_i - \hbar\omega) \tag{23}$$

7.4.5 Final state is d_{xy}

$$\sigma^Q(\hat{\epsilon}, \hat{k}) \propto \alpha\hbar\omega k^2 \left(\frac{2}{\sqrt{5}} + \frac{1}{7\sqrt{5}}(35\sin^2\theta\cos^2\theta - 4) + \sqrt{5}\sin^2\theta\cos^2\theta\sin 4\phi\right) \xi_{xy}\delta(E_f - E_i - \hbar\omega) \tag{24}$$

7.4.6 Final state is d_{xz},d_{yz}

$$\sigma^Q(\hat{\epsilon}, \hat{k}) \propto \alpha\hbar\omega k^2 \left(\frac{2}{\sqrt{5}} - \frac{4}{7\sqrt{5}}(35\sin^2\theta\cos^2\theta - 4)\right) \xi_{xz}\delta(E_f - E_i - \hbar\omega) \tag{25}$$

References

[1] Shang-Di Mo and W Y Ching. Electronic and optical properties of three phases of titanium dioxide: Rutile, anatase, and brookite. *Physical Review B*, 51(19):13023–13032, 1995.

[2] J R DeVore. Refractive Indices of Rutile and Sphalerite. *JOSA*, 41(6):416–419, 1951.

[3] Matts Björck and Gabriella Andersson. GenX: An extensible X-ray reflectivity refinement program utilizing differential evolution. *Journal of Applied Crystallography*, 40(6):1174–1178, 2007.

[4] Jens Als-Nielsen and Des McMorrow. *Elements of Modern X-ray Physics*. John Wiley & Sons, 2011.

[5] C Brouder, J P Kappler, and E Beaurepaire. Theory and application of angle resolved X-ray absorption spectra. *Conference Proceedings - 2nd European Conference on Progress in X-ray Synchrotron Radiation Research*, 25:19–22, 1990.

[6] Christian Brouder, Amélie Juhin, Amélie Bordage, and Marie-Anne Arrio. Site symmetry and crystal symmetry: a spherical tensor analysis. *Journal of Physics: Condensed Matter*, 20(45):455205–16, 2008.
